# Improving Methodological Quality in Meta-Analyses of Athlete Pain Interventions: An Overview of Systematic Reviews

**DOI:** 10.3390/healthcare13192508

**Published:** 2025-10-02

**Authors:** Saul Pineda-Escobar, Cristina García-Muñoz, Olga Villar-Alises, Javier Martinez-Calderon

**Affiliations:** 1 Departamento de Fisioterapia, Universidad de Sevilla, 41009 Sevilla, Spain; saulpinedaescobar@gmail.com (S.P.-E.); olgavillar711@gmail.com (O.V.-A.); jmcalderon@us.es (J.M.-C.); 2 CTS 1110: Uncertainty, Mindfulness, Self, and Spirituality (UMSS) Research Group, 41009 Andalusia, Spain; 3 Departamento Ciencias de la Salud y Biomédicas, Universidad Loyola Andalucía, 41704 Sevilla, Spain; 4 Instituto de Biomedicina de Sevilla-IBiS, Hospitales Universitarios Virgen del Rocío y Macarena, CSIC, Universidad de Sevilla, 41013 Sevilla, Spain

**Keywords:** pain, athletes, systematic review, meta-analysis, methodological quality, sports rehabilitation

## Abstract

Background: Pain is a disabling issue in athletes, with significant impact on performance and career longevity. Many randomized clinical trials (RCTs) have explored interventions to reduce pain, leading to multiple systematic reviews with meta-analysis, but their methodological rigor and clinical applicability remain unclear. Objective: To provide an overview of systematic reviews with meta-analysis on interventions aimed at alleviating pain intensity in athletes, identifying knowledge gaps and appraising methodological quality. Methods: CINAHL, Embase, Epistemonikos, PubMed, Scopus, SPORTDiscus, and Cochrane Library were searched from inception to February 2025. Systematic reviews with meta-analysis of RCTs evaluating interventions to manage pain in athletes were considered. Athletes without restrictions in terms of sports, clinical, and sociodemographic characteristics were included. Overlap between reviews was calculated using the corrected covered area. Results: Twelve systematic reviews met inclusion criteria. Physical exercise modalities (e.g., gait retraining, hip strengthening), acupuncture, photo biomodulation, and topical medication showed potential benefits in reducing pain intensity. Other interventions, such as certain manual therapy techniques, platelet-rich plasma, or motor imagery, did not show consistent effects. All reviews focused solely on pain intensity, with minimal stratification by sport or clinical condition which may affect the extrapolation of meta-analyzed findings to the clinical practice. Methodological quality was often low, with flaws in reporting funding sources, lists of excluded studies, and certainty of evidence (was mostly rated as low/very low). Overlap was variable across the interventions. Conclusions: Given low/sparse certainty and minimal sport-specific analyses, no strong clinical recommendations can be made; preliminary signals favor proximal hip strengthening, gait retraining, photo biomodulation (acute soreness), and topical NSAIDs pending higher-quality syntheses. Future reviews should consider mandatory GRADE; pre-registered protocols; sport- and condition-specific analyses; and core outcome sets including multi-dimensional pain.

## 1. Introduction

Pain in sports represents a significant issue that directly affects both the health and athletic careers of athletes [1], with high prevalence rates in different sports populations. For instance, chronic low back pain may affect between 18% and 65% of athletes, with potential consequences for sports performance and causing an early sports retirement in some cases [2]. Knee pain may appear in up to 60% of basketball players in an observational study on collegiate basketball players in Canada [3].

Pain often appears as a core symptom in sports injuries mainly related to the musculoskeletal system (e.g., spine or upper limbs) [4]. In some cases, these injuries may lead to athletes developing chronic health conditions such as chronic ankle instability [5], and thus, acute pain may move to chronic pain. Musculoskeletal injuries and pain are an important economic burden in sports [6]. In collegiate athletes, annual rehabilitation costs are estimated to exceed $1000 per athlete [7], and the average cost per muscle injury approximately reaches $450,000 in professional athletes playing football [8]. Furthermore, pain intensity is often the pain dimension explored in sports, but other pain dimensions such as pain sensitivity and the role of biopsychosocial factors in the management of sports injuries has been recently explored in athletes [9,10].

These factors have underlined the complexity of pain management, and thus, it seems plausible to seek the most robust evidence to decide what interventions may be most useful to alleviate pain in specific sports and individual athletes. Currently, we have available a large number of systematic reviews that have supported the use of pain-related medication, autologous therapies (e.g., platelet-rich plasma), stretching routines, and physical exercise-based interventions to manage pain in athletes [11,12,13]. However, current systematic reviews in this field may show some methodological shortcomings that should be highlighted and critically analyzed (e.g., heterogeneity in populations, inconsistent outcome measures, lack of GRADE use). It is especially essential to critically evaluate the methodological quality of meta-analyses due to their direct influence on evidence-based clinical practice (e.g., in the development of clinical practice guidelines).

Currently, the proliferation in the number of systematic reviews in this field may show that the topic is of great interest to the sports community, which has led to the publication of multiple clinical trials in recent years. This has permitted the development and publication of numerous systematic reviews that have meta-analyzed the effects of different interventions to alleviate pain in athletes [13,14]. We believe it is time to develop an overview of systematic reviews that sheds light on which meta-analyzed interventions may be most effective in reducing pain in athletes, in which sports these interventions may work best, and what the certainty is of these meta-analyses.

The objective of this study is to develop an overview of systematic reviews with meta-analysis to critically appraise the methodological quality and clinical applicability of systematic reviews with meta-analysis on pain interventions in athletes. This work will provide sports health professionals with a critically appraised body of evidence in a single article that we hope can support informed clinical decision-making.

## 2. Materials and Methods

The PRIOR statement (Preferred Reporting Items for Overviews of Reviews) and the PRISMA 2020 statement (Preferred Reporting Items for Systematic Reviews and Meta-Analyses) for abstracts were followed [15,16]. The review protocol was prospectively registered on the Open Science Framework: https://osf.io/sn7ah (accessed on 20 February 2025).

### 2.1. Deviations from the Review Protocol

The following deviations were carried out to develop more homogenous analyses and reach more firm and clinical conclusions. Only meta-analyses focused on interventions were eventually considered. Finally, we opened this overview to all types of interventions to show the whole picture of what meta-analyses have been published considering the effects of a specific intervention to alleviate pain. The degree of overlap was calculated when two meta-analyses explored the same or related interventions (e.g., different types of manual therapy).

### 2.2. Data Sources and Search Strategies

The following e-databases were searched from inception to 20 February 2025: CINAHL (via EBSCOhost, Ipswich, MA, USA), Embase, Epistemonikos, PubMed, Scopus, SPORTDiscus (via EBSCOhost), and the Cochrane Library. We also performed manual searches in those overviews of reviews, scoping reviews, and review protocols that were retrieved during search strategies and were related to our topic. Search filters by type of document were included when possible (Supplementary File S1). An example of the full search strategy is reported below, whereas all full search strategies are reported in Supplementary File S1. The following search was built for PubMed, but all the search strategies can be found in Supplementary File S1: (athlete* [tiab] OR player* [tiab]) AND (pain [tiab]) AND (systematic-review [title] OR meta-analysis [title] OR metaanalysis [title] OR meta-analyses [title] OR metaanalyses [title] OR meta-review [title] OR meta-analytic-review [title] OR overview-of-systematic [title] OR overview-of-reviews [title] OR umbrella-review [title] OR scoping review [title]).

### 2.3. Eligibility Criteria

The PICOS (Population, Intervention, Comparison, Outcome, Study design) framework was used to develop the eligibility criteria [17].

P: Athletes without restrictions in terms of sports, clinical, and sociodemographic characteristics. However, it is important to underline that there are sports which involve pain-inducing techniques, and first and foremost, pain signals the success or failure of a certain technique, especially in contact sports.

I: No restrictions were imposed regarding the type of intervention.

C: No restrictions were imposed in terms of the comparison groups.

O: Any dimension of pain (e.g., pain intensity or pain sensitivity).

S: Systematic review with meta-analysis of randomized clinical trials published in peer-reviewed journals. Any type of randomized clinical trial was considered (e.g., pilot randomized clinical trial).

The following exclusion criteria were considered: (1) meta-analyses combining studies exploring different types of population (e.g., athletic and non-athletic populations); (2) the full text was not available after requesting via email to the corresponding author.

### 2.4. Study Selection

The study selection was independently performed by two co-authors (JMC and SPE). One co-author (SPE) used Zotero 6.0.36 Citation Management Software to handle the references retrieved from e-databases. This co-author deleted duplicates and evaluated titles and abstracts. Then, two co-authors (JMC and SPE) analyzed full texts of those abstracts that seemed to be eligible or those studies where abstracts were unavailable. The percentage of agreement between these co-authors was calculated. This percentage was obtained considering the number of items rated with the same score before pooling the results of their independent assessments. The percentage of agreement was 92.86% in the first round and 100% after discussing the differences between them. A third reviewer was not necessary in this section. Supplementary File S2 shows all excluded studies during full text analysis and the reason for their exclusion.

### 2.5. Methodological Quality Assessment

Two co-authors (OVA and SPE) applied independently the AMSTAR 2 checklist (A MeaSurement Tool to Assess systematic Reviews 2) to assess the methodological quality of systematic reviews [18]. AMSTAR 2 is composed of 16 items, and each item can be scored as “Yes”, “Partially yes”, or “No”. No overall score is recommended by the authors of this checklist, but seven items are recommended as critical domains (items 2, 4, 7, 9, 11, 13, and 15) [18]. These items are related to potential protocol deviations (item 2), search strategies (item 4), reporting a list of excluded studies (item 7), evaluation risk of bias (item 9), methods to conduct meta-analysis (item 11), possible implications of risk of bias (item 13), and publications bias (item 15).

The percentage of agreement between these co-authors was calculated. This percentage was obtained considering the number of items rated with the same score before pooling the results of their independent assessments. The percentage of agreement was 97.9% in the first round and 100% after discussing the differences between them. A third reviewer was not necessary in this section.

### 2.6. Data Extraction

Two co-authors (JMC and SPE) independently developed the data extraction process. One co-author (SPE) extracted from each review the following information: (1) study and year of publication, (2) objective original review, (3) number total of participants, (4) population characteristics, (5) number total of randomized clinical trials, (6) outcomes analyzed in this overview, (7) type of intervention, (8) intervention characteristics, (9) types of control groups. Then, two co-authors (JMC and SPE) independently extracted from each review pooled findings and certainty of evidence using the Grading of Recommendations, Assessment, Development and Evaluation (GRADE) system. The percentage of agreement between these co-authors was calculated. This percentage was obtained considering the number of items rated with the same score before pooling the results of their independent assessments. The percentage of agreement was 100%.

### 2.7. Data Analysis

The main results regarding the meta-analyses of interest are shown in Table 1 and reported by type of intervention and outcome measure in the main text (e.g., effects of eccentric exercise on pain intensity). Due to the large number of modalities that have been found, we have categorized several interventions under the umbrella term “manual therapy techniques”, where we have included interventions such as dry needling, stretching routines, or massage.

In addition, one co-author (JMC) calculated the degree of overlap between meta-analyses of interest. This overlap was only calculated if at least two systematic reviews meta-analyzed the same type of intervention (e.g., eccentric exercise) and outcome (e.g., pain intensity). Firstly, JMC matrices of evidence were developed. These matrices are reported in Supplementary Files S3–S6. Afterward, JMC calculated the corrected covered area (CCA), that is, the covered area after original studies are removed the first time they are counted [31]. The formula is obtained using the following:

(I) N: The total number of original studies (including duplicates) in the meta-analyses of interest (the sum of all checked boxes in the citation matrix).
(II) r: The number of original studies without accounting for duplicates.
(III) c: The number of systematic reviews included in the matrix of evidence.

The CCA is needed to know the degree of overlap between meta-analyses of interest and permits to us to classify the degree of overlap as slight (CCA 0–5%); moderate (CCA 6–10%); high (CCA 11–15%); or very high (CCA > 15%) [31]. Finally, one co-author (CGM) developed a bar plot to depict the degree of overlap between meta-analyses of interest.

## 3. Results

**Figure 1:**
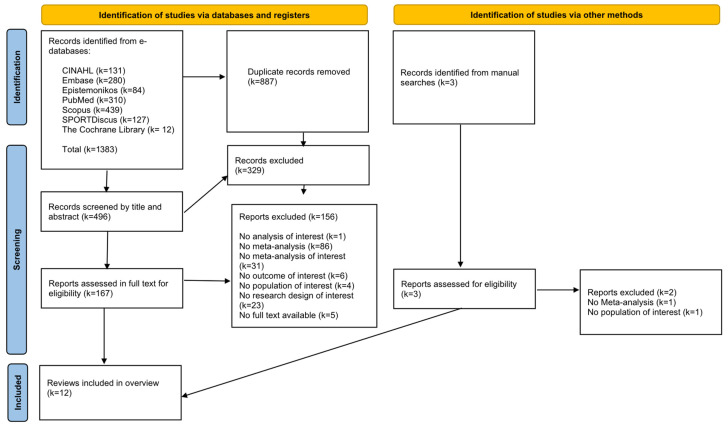
Flow diagram PRISMA 2020.

A total of 1383 references were retrieved from e-databases. Of them, 496 titles and abstracts were evaluated after removing duplicates. Then, 167 references were analyzed at full text, and 12 studies were eventually included (Figure 1). In addition, three references were manually found and all of them were excluded.

### 3.1. The Degree of Overlap Between Meta-Analyses

**Figure 2:**
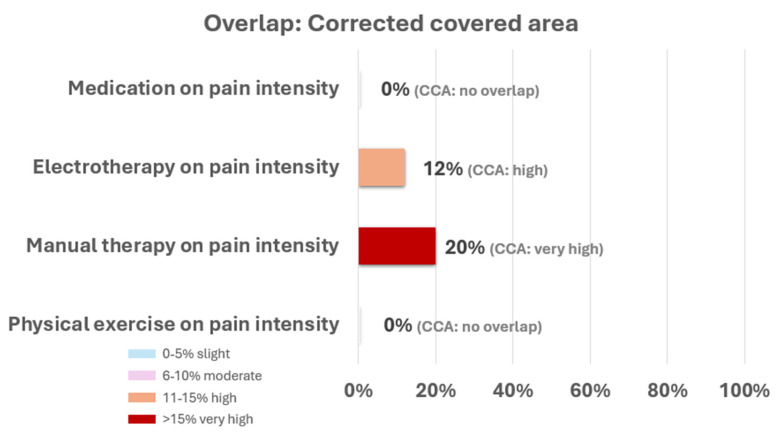
Percentage of overlap using the corrected covered area of the different outcomes.

The degree of overlap was very high for those meta-analyses evaluating the effects of manual therapy techniques on pain intensity (CCA = 20%). There was high overlap for those meta-analyses analyzing electrotherapy on pain intensity (CCA = 12%). No overlap was found among the meta-analyses evaluating the effects of physical exercise on pain intensity (CCA = 0%) or meta-analyses evaluating the effects of medication on pain intensity (CCA = 0%). The rest of the interventions were not analyzed because only one meta-analysis evaluates them (e.g., acupuncture). Figure 2 represents the overlap results described above. Matrices of evidence and CCA calculations are reported in Supplementary Files S3–S6.

### 3.2. Methodological Quality Assessment (AMSTAR 2)

The assessment of each review is reported in Table 2. Some systematic reviews showed methodological flaws related to the selection of the study designs for inclusion in the review (item 3). In addition, some systematic reviews did not report a list of excluded studies and a justification for their exclusions (item 7). Finally, it is also important to underline that the reporting of the sources of funding for primary research included in these reviews was not included (item 10).

### 3.3. Pooled Findings

All included reviews only meta-analyzed pain intensity, and the rest of pain dimensions (e.g., pain sensitivity) could not be evaluated in this overview. The included reviews were focused on physical exercise programs (e.g., eccentric exercise), manual therapy techniques (e.g., stretching), acupuncture, electrotherapy, motor imagery, or medication (e.g., oral medication). The main characteristics of the systematic reviews are reported in Table 1.

### 3.4. Effects of Physical Exercise Programs on Pain Intensity

#### 3.4.1. Proximal Hip Strengthening Exercise

Proximal hip strengthening exercises showed significant reduction in pain intensity compared to control in recreational runners, although the meta-analysis showed high heterogeneity (SMD 1.80; 95%CI 1.21 to 2.38; I^2^ 79%; k = 2; N = 68) [27]. Hip muscle strengthening exercises also showed significant reduction in pain intensity compared to control in athletes from other sports (e.g., soccer), although the meta-analysis showed high heterogeneity (MD 2.45; 95%CI 1.11 to 3.79; I^2^ 65%; k = 2; N = 88) [24].

#### 3.4.2. Gait Retraining

Gait retraining showed significant reduction in pain intensity compared to control in recreational runners, and the meta-analysis showed low heterogeneity (SMD 3.84; 95%CI 2.70 to 4.98; I^2^ 26%; k = 2; N = 40) [27].

#### 3.4.3. Eccentric Exercise

No differences were found between eccentric exercise and different control groups (e.g., usual training) regarding the reduction in pain intensity in athletes (MD −2.00; 95%CI −5.14 to 1.15; I^2^ UR; k = 3; N = 68) However, the meta-analysis did not specify the type of sports analyzed and the I-square was not reported [19].

#### 3.4.4. Different Exercise Modalities Combined in the Same Meta-Analysis

Different exercise modalities (e.g., core spinal stabilization) combined in the same meta-analysis showed significant reduction in pain intensity compared to control in athletes from different sports (e.g., hockey and cricket), although the meta-analysis showed high heterogeneity (MD −1.65; 95%CI −2.74 to −0.55; I^2^ 91%; k = 3; N = UR) [30].

### 3.5. Effects of Manual Therapy Techniques on Pain Intensity

Different manual therapy techniques (e.g., stretching, myofascial release, or dry needling) combined in the same meta-analysis showed no differences in alleviating pain intensity compared to control in athletes from different sports, although the meta-analysis showed high heterogeneity (SMD 0.02; 95%CI −1.45 to 1.48; I^2^ 93%; k = 4; N = 147) [22].

In addition, no differences between stretching exercises and control were found in athletes from different sports, and the meta-analysis showed no heterogeneity (MD −0.51; 95%CI −1.14 to 0.13; I^2^ 0%; k = 2; N = 61) [20].

### 3.6. Effects of Acupuncture on Pain Intensity

Acupuncture showed significant reduction in pain intensity compared to control in athletes. However, the meta-analysis did not specify the type of sport analyzed, and the heterogeneity was high (MD −1.31; 95%CI −1.70 to −0.93; I^2^ 93%; k = 15; N = 1.131) [23].

### 3.7. Effects of Electrotherapy on Pain Intensity

Photo biomodulation showed significant reduction in pain intensity compared to control in athletes from different sports (SMD 1.03; 95%CI 0.43 to 1.63; I^2^ UR; k = 5; N = UR) [26].

In addition, photo biomodulation showed significant reduction in pain intensity compared to control in soccer players at post-exercise (SMD −1.54; 95%CI −2.90 to −0.19; I^2^ 86%; k = 4; N = 100), with the meta-analysis showing high heterogeneity [25].

On the other hand, no differences between photo biomodulation and control were found in soccer players for pain intensity at 24 h follow-up (SMD −0.27; 95%CI −0.87 to 0.32; I^2^ 25%; k = 3; N = 68), 48 h follow-up (SMD 0.31; 95%CI −0.48 to 1.09; I^2^ 53%; k = 3; N = 68), and 96 h follow-up (SMD 0.46; 95%CI −0.40 to 1.31; I^2^ 59%; k = 3; N = 68), with moderate heterogeneity in the meta-analyses [25].

### 3.8. Effects of Medication on Pain Intensity

Topical medication showed significant reduction in pain intensity compared to control in athletes from different sports, although the meta-analysis showed high heterogeneity (g −0.64; 95%CI −0.89 to −0.39; I^2^ 71%; k = 8; N = 994,) [28].

On the other hand, platelet-rich plasma (injection and gel) (ES −0.21; 95%CI −2.29 to 1.87; I^2^ 0%; k = 5 N = UR) [21] and oral medication (g −0.22; 95%CI −0.60 to 0.17; I^2^ 55%; k = 5; N = 286) [28] showed a lack of significant differences for reducing pain intensity compared to control in athletes from different sports, and the meta-analysis showed no heterogeneity (platelet-rich plasma) and moderate heterogeneity (oral medication).

### 3.9. Effects of Motor Imagery on Pain Intensity

No differences between motor imagery and control were found for reducing pain intensity in athletes from different sports (MD −1.57; 95%CI −3.60 to 0.46; I^2^ 50%; k = 3; N = 50), and the meta-analysis showed moderate heterogeneity [29].

### 3.10. Certainty of Evidence Assessment (GRADE)

Four out of twelve reviews used GRADE for assessing the certainy of evidence of the meta-analysis included in this overview. Overall, the certainy of evidence in these meta-analyses ranged from low to very low. Table 3 shows the GRADE assessment for each review.

## 4. Discussion

This overview of systematic reviews aimed to show the pooled effects on the effectiveness of different interventions to modulate pain in athletes from different sports. After analyzing 12 systematic reviews with meta-analysis, we found that some physical exercise modalities (e.g., gait retraining), acupuncture, photo biomodulation, and topical medication could alleviate pain intensity in athletes. However, the results should be interpreted with caution because important methodological problems and gaps in knowledge were detected. They should be corrected and completed in the future, given the importance of pain in sports rehabilitation.

First, the results found in this overview, although informative, are extremely difficult to extrapolate to clinical practice. This is mainly because only two of the 12 systematic reviews developed meta-analyses of interest by type of sport [25,27]. The remaining meta-analyses combined different sports or did not specify the type of sport meta-analyzed. Finally, we could only find specific data for runners and soccer players, and this information was related to physical exercise programs (gait retraining and strengthening exercises) and photo biomodulation, respectively. In this context, a huge gap in knowledge remains in this field regarding the extrapolation of data from these meta-analyses to daily clinical practice.

Second, the objective of this overview was to synthesize meta-analyses that evaluated interventions to manage pain in athletes. However, the included meta-analyses only explored one dimension of pain: pain intensity. The importance of other pain dimensions such as pain sensitivity has been recently supported in sports populations by a systematic review with meta-analysis. This review found that athletes may show higher pain tolerance than non-athlete populations [9]. In addition, another review has underlined the relevance of considering multiple biopsychosocial factors when sports injuries are explored [10]. Particularly in athletes with pain, a recent study showed that athletes and non-athletes both with low back pain reported factors such as fear-avoidance and endurance-related responses (e.g., thought suppression) [32]. Despite the importance of previous evidence regarding the multidimensional nature of pain in athletes, sports science shows some gaps in knowledge considering aspects such as pain mechanisms, other pain dimensions rather than pain intensity, and interventions to manage those pain dimensions [33].

Third, we found enormous variability among the included reviews regarding the musculoskeletal injuries analyzed. Conditions such as knee osteoarthritis, patellofemoral pain syndrome, chronic patellar tendinopathy, glenohumeral internal rotation deficit, groin pain, and low back pain were specifically meta-analyzed [17,18,19,24,27,31]. However, we found a significant gap in knowledge regarding pooled effects for pain modulation in clinical health conditions such as ankle sprains, Achilles tendinopathy, stress fractures, lumbar spine injuries in rowers, or shoulder impingement in swimmers.

Finally, only 4 of the 12 reviews used the GRADE approach to assess the certainty of the meta-analyzed evidence [20,22,24,29]. This is a critical point, as in most meta-analyses, we do not know how the risk of bias, inconsistency, imprecision, indirectness, or publication bias could have affected the results. Furthermore, the lack of use of GRADE could negatively impact the development of new clinical guidelines on this topic or affect policymakers’ lack of certainty grading.

This is even more important when we note that in those reviews where GRADE was used, the certainty of the evidence was mostly low or very low, mainly due to problems related to risk of bias, inconsistency, imprecision, and high heterogeneity observed in several meta-analyses (e.g., diverse populations, multiple and varied interventions, inconsistent comparators). This fits with the reasons we highlighted above and why we have asked readers to be cautious when analyzing the results of this overview.

### 4.1. Clinical Implications

The trend in many meta-analyses shows that some interventions (e.g., some forms of physical exercise) could have positive effects in alleviating pain intensity. For example, gait retraining proven effective in recreational runners cannot be directly extrapolated to swimmers or rowers because of fundamental biomechanical differences. Likewise, hip strengthening programs that benefit soccer players may not translate to overhead athletes such as tennis players, where shoulder-dominant kinetics predominate. However, given the methodological issues that have been highlighted above, current evidence does not support strong recommendations. In this context, we recommend sports clinicians to be cautious with the use of conclusions of previous systematic reviews in this field and encourage them to be critical with the current evidence on this topic. When interpreting our findings, clinicians should consider the certainty of evidence assessed with GRADE. For example, given the very low certainty supporting hip strengthening programs, these interventions should be applied cautiously and monitored on an individual basis until higher-quality trials confirm their effectiveness. Likewise, the low certainty for photo biomodulation means it should not be implemented as routine care without further supporting evidence. When certainty of evidence is low, athletic trainers and team physicians should prioritize interventions with the most consistent favorable signals and minimal risk, such as supervised strengthening or gait retraining programs for runners, while considering athlete preference and ease of implementation. Shared decision-making and close monitoring of outcomes are essential until stronger sport-specific evidence becomes available.

### 4.2. Future Research

Our main recommendation is to update systematic reviews on this topic, primarily with the goal of developing new analyses that allow for greater extrapolation of results to clinical practice. Mainly, our recommendation is directed toward developing analyses stratified by competition level, injury type, and include cost-effectiveness analyses.

Furthermore, we encourage sports researchers to expand their knowledge by considering the effects of different interventions to manage different pain dimensions’ intensity in clinical health conditions such as ankle sprains and Achilles tendinopathy, stress fractures, lumbar spine injuries in rowers, or shoulder impingement in swimmers. Future systematic reviews should adopt standardized pain outcome sets that go beyond pain intensity, such as pain sensitivity, interference, and psychosocial dimensions, and should clearly report the funding sources of included studies to enhance transparency and reduce risk of bias.

### 4.3. Stregthns and Limitations

The main strengths of this overview were the methodological innovations of this study, including aspects such as OSF registration, CCA analysis, and adherence to PRIOR.

However, we acknowledge that the inclusion criteria were strict and that only meta-analyses evaluating athletic populations were considered. Therefore, meta-analyses that combined non-athletic and athletic populations may have been missed. We did not analyze the meta-analyzed findings by clinical health conditions due to the enormous variability in the data found regarding type of sport and interventions. However, this data can be found in Table 1. The inclusion criterion “athletes without restriction” is broad and risks conflating elite, recreational, and clinical populations, which may reduce the specificity of our conclusions. Furthermore, we acknowledge that including some meta-analyses under the umbrella term “manual therapy techniques” may affect the extrapolation of the results into clinical practice. Unfortunately, a meta-analysis combined different manual therapy techniques and thus, we maintained this term in this study. Finally, as with any evidence synthesis, our overview may be subject to publication bias. Although we searched multiple databases and performed manual checks, relevant gray literature or unpublished studies might have been missed, which could influence the comprehensiveness of the evidence base.

## 5. Conclusions

While some interventions may alleviate pain intensity in athletes across various sports and with different clinical conditions, the current state of most meta-analyses (mainly low/very low evidence when GRADE was used) prevents the formulation of strong recommendations that can be adopted in clinical practice. Mandatory protocol registration, GRADE assessment, and analyses stratified by sport/condition should be a priority in future reviews. Coaches, athletic trainers, and policymakers should know that there are other pain dimensions that have not been explored in previous reviews rather than pain intensity, and no robust meta-analyzed data are available for most of the sports (e.g., swimming, cricket, basketball, or hockey).

## Figures and Tables

**Table 1:** Characteristics of the included systematic reviews.

Study and Year of Publication	Main Characteristics of Meta-analyses Analyzed	Intervention Characteristics	Control Groups
Araya Quintanilla et al., 2012 [19]	Population: athletes; gender: male and female; age: 22–29 years. Number of randomized clinical trials: 3 Outcome analyzed in this overview: pain intensity. Clinical condition: chronic patellar tendinopathy. Type of intervention: eccentric exercise.	Eccentric exercise (declined platform 25°)	Eccentric exercise conventional (not declined) Usual training
Ceballos-Laita et al., 2024 [20]	Population: athletes (volleyball, tennis, swimming, baseball); gender: male and female; age: 16–22 years. Number of randomized clinical trials: 2 Outcome analyzed in this overview: pain intensity. Clinical condition: glenohumeral internal rotation deficit. Type of intervention: stretching.	Stretching (sleeper stretch; sleeper stretch + standard care)	Standard care
Gholami et al., 2016 [21]	Population: Athletes; gender: male and female; age: >18 years. Number of randomized clinical trials: 5 Outcome analyzed in this overview: pain intensity. Clinical condition: patellar tendinopathy, lateral elbow epicondylitis, rotator cuff tendinopathy, knee. Type of intervention: platelet-rich plasma (injection and gel).	Platelet-rich plasma	Corticosteroid Autologous whole blood Dry needling Saline
Jiménez-del-Barrio et al., 2022 [22]	Population: athletes (volleyball, tennis, swimming, handball, baseball, water polo, squash); gender: male and female; age: 18–28 years. Number of randomized clinical trials: 4 Outcome analyzed in this overview: pain intensity. Clinical condition: glenohumeral internal rotation deficit and chronic shoulder pain. Type of intervention: stretching, manual therapy, and dry needling teres mayor.	Stretching Anterior–posterior mobilization grade III Passive glenohumeral rotation with clam shell bridging Dry needling teres major	Manual contact Modified sleeper stretch
Jin et al., 2024 [23]	Population: athletes; gender: UR; age; UR. Number of randomized clinical trials: 15 Outcome analyzed in this overview: pain intensity. Clinical condition: knee osteoarthritis. Type of intervention: acupuncture.	Contralateral cross-acupuncture alone Contralateral cross-acupuncture + joint loosening or oral drug treatment or seedling medicine pain patch or lidocaine local infiltration anesthesia	Knee local acupuncture. Electro-acupuncture local + joint loosening Oral drug treatment Lidocaine local infiltration anesthesia
Lahuerta-Martín et al., 2023 [24]	Population: athletes (soccer, rugby, squash, running, hockey, skating, and other sports); gender: male and female; age: 21–35 years. Number of randomized clinical trials: 2 Outcome analyzed in this overview: pain intensity. Clinical condition: groin pain. Type of intervention: physical exercise.	Hip muscle strengthening	Passive physical therapy plus return to running program
Luo et al., 2022 [25]	Population: athletes (college athletes and soccer players); gender: male and female; age: 15–35 years. Number of randomized clinical trials: 4 Outcome analyzed in this overview: pain intensity. Clinical condition: muscle soreness. Type of intervention: low-level laser therapy.	Photo biomodulation	Sham photo biomodulation
Morgan et al., 2024 [26]	Population: athletes (volleyball, handball, track and field, college and recreational athletes); gender: male and female (Takenori et al.); age: mean age of 24 years. Number of randomized clinical trials: 5 Outcome analyzed in this overview: pain intensity. Clinical condition: groin pain, proximal hamstring tendinopathy, patella tendinitis, meniscal injuries, Achilles tendinopathy, ankle sprain, navicular fracture, plantar fasciitis, TFCC injury, proximal thumb avulsion, elbow medial collateral ligament sprain, shoulder arthroscopic surgery, infraspinatus muscle injury, deltoid muscle injury, shoulder periarthritis, low back pain, lumbar facet arthritis, spondylolysis. Type of intervention: photo biomodulation.	Photo biomodulation Photo biomodulation (LLLT) + eccentric strengthening Photo biomodulation (LLLT) + trigger band technique + medical treatment Photo biomodulation (HPLT)	Placebo Placebo photo biomodulation (LLLT) + eccentric strengthening Placebo photo biomodulation (LLLT) + trigger band technique + medical treatment Conventional physical treatment
Neal et al., 2016 [27]	Population: athletes (recreational runners); gender: male and female; age: 18–50 years. Number of randomized clinical trials: 4 Outcome analyzed in this overview: pain intensity. Clinical condition: patellar femoral pain. Type of intervention: strengthening exercise and running gait retraining.	Proximal (hip) strengthening exercise Running gait retraining	Pre-intervention
Nudo et al., 2023 [28]	Population: recreational and competitive athletes (kayakers, soccer, handball, basketball, karate, and other sports); gender: male and female; age: 18–58 years. Number of randomized clinical trials: 13 Outcome analyzed in this overview: pain intensity. Clinical condition: sprain, strain, contusion, Achilles tendinopathy, chronic patellar tendinopathy, wrist extensor tenosynovitis, acute hamstring injuries. Type of intervention: topical or oral medication.	Oral or Topical Medication: piroxicam 40 mg, ibuprofen 600 mg, ibuprofen 200 mg, diclofenac 140 mg diclofenac 25 mg, naproxen 500 mg, meclofenamate, escin 1%/2%, dimethylammonium salicylate 5%, heparin 500 IU	Placebo
Plakoutsis et al., 2022 [29]	Population: athletes; gender: male and female; age: 18–50 years. Number of randomized clinical trials: 3 Outcome analyzed in this overview: pain intensity. Clinical condition: lower limb injuries (ankle sprain, grade II, ACL reconstructive surgery). Type of intervention: motor imagery.	Relaxation and imagery Relaxation and guided imagery Kinesthetic imagery	Physical therapy program
Thornton et al., 2021 [30]	Population: athletes (hockey and cricket); male, female, and unspecified; age: 15–72 years. Number of randomized clinical trials: 3 Outcome analyzed in this overview: pain intensity. Clinical condition: low back pain. Type of intervention: physical exercises.	Periodized resistance training Core spinal stabilization	Allowed to continue their recreational activity Conventional exercises, physiotherapy, strengthening exercises

Note: UR: unreported; LLLT: low-level laser therapy; HPLT: high-power laser therapy; TFCC: triangular fibrocartilage complex.

**Table 2:** The methodological quality of systematic reviews (AMSTAR 2).

Author(s)	1	2	3	4	5	6	7	8	9	10	11	12	13	14	15	16
Araya Quintanilla et al., 2012 [19]																
Ceballos-Laita et al., 2024 [20]																
Gholami et al., 2016 [21]																
Jiménez-del-Barrio et al., 2022 [22]																
Jin et al., 2024 [23]																
Lahuerta-Martín et al., 2023 [24]																
Luo et al., 2022 [25]																
Morgan et al., 2024 [26]																
Neal et al., 2016 [27]																
Nudo et al., 2023 [28]																
Plakoutsis et al., 2022 [29]																
Thornton et al., 2021 [30]																

Note: Answers: red color: No, yellow color: Partially yes, green color: Yes. Items: AMSTAR 1: Did the research questions and inclusion criteria for the review include the components of PICO? AMSTAR 2: Did the report of the review contain an explicit statement that the review methods were established prior to the conduct of the review and did the report justify any significant deviations from the protocol? AMSTAR 3: Did the review authors explain their selection of the study designs for inclusion in the review? AMSTAR 4: Did the review authors use a comprehensive literature search strategy? AMSTAR 5: Did the review authors perform study selection in duplicate? AMSTAR 6: Did the review authors perform data extraction in duplicate? AMSTAR 7: Did the review authors provide a list of excluded studies and justify the exclusions? AMSTAR 8: Did the review authors describe the included studies in adequate detail? AMSTAR 9: Did the review authors use a satisfactory technique for assessing the risk of bias in individual studies that were included in the review? AMSTAR 10: Did the review authors report on the sources of funding for the studies included in the review? AMSTAR 11: If meta-analysis was performed, did the review authors use appropriate methods for statistical combination of results? AMSTAR 12: If meta-analysis was performed, did the review authors assess the potential impact of risk of bias in individual studies on the results of the meta-analysis or other evidence synthesis? AMSTAR 13: Did the review authors account for risk of bias in individual studies when interpreting/discussing the results of the review? AMSTAR 14: Did the review authors provide a satisfactory explanation for, and discussion of, any heterogeneity observed in the results of the review? AMSTAR 15: If they performed quantitative synthesis, did the review authors carry out an adequate investigation of publication bias (small study bias) and discuss its likely impact on the results of the review? AMSTAR 16: Did the review authors report any potential sources of conflicts of interest, including any funding they received for conducting the review?

**Table 3:** Certainty of evidence assessment (GRADE).

Review	GRADE Assessment	Reasons
Ceballos-Laita et al., 2024 [20] (stretching)	Very low evidence	Serious risk of bias, serious indirectness, and serious imprecision
Jiménez-del-Barrio et al., 2022 [22] (different manual therapy techniques combined in the same meta-analysis)	Moderate evidence	Serious imprecision
Lahuerta-Martín et al., 2023 [24] (hip streghthening exercises)	Very low evidence	Serious risk of bias, serious inconsistency, and serious imprecision
Plakoutsis et al., 2022 [29] (motor imagery)	Low evidence	Serious inconsistency and serious imprecision

## Data Availability

No extra data is available.

## Supplementary Materials

The following supporting information can be downloaded at: https://www.mdpi.com/article/10.3390/healthcare13192508/s1, Supplementary File S1: Search strategies; Supplementary File S2 Excluded studies list; Supplementary File S3: Overlap exercise; Supplementary File S4: Overlap manual therapy; Supplementary File S5: Overlap electrotherapy; Supplementary File S6: Overlap medication.
