# Supplementary material for: Improving Methodological Quality in Meta-Analyses of Athlete Pain Interventions: An Overview of Systematic Reviews
Source: Healthcare (Basel). 2025 Oct 2;13(19):2508. doi: 10.3390/healthcare13192508 (PMC12524677; doi:10.3390/healthcare13192508)
Supplement: Supplementary file 1 [file healthcare-13-02508-s001.zip › 1 PRIOR checklist.pdf]

PRIOR Checklist

(Gates M, Gates A, Pieper D, et al. Reporting guideline for overviews of reviews of healthcare interventions: development of the PRIOR statement. ***BMJ*** **2022;378:e070849. doi:10.1136/bmj-2022-070849.)**

**Section** Topic	**#**	**Item**	**Location reported**
**TITLE**			
Title	1	Identify the report as an overview of reviews.	1
**ABSTRACT**			
Abstract	2	Provide a comprehensive and accurate summary of the purpose, methods, and results of the overview of reviews.	3
**INTRODUCTION**			
Rationale	3	Describe the rationale for conducting the overview of reviews in the context of existing knowledge.	4-5
Objectives	4	Provide an explicit statement of the objective(s) or question(s) addressed by the overview of reviews.	5
**METHODS**			
Eligibility criteria	5a	Specify the inclusion and exclusion criteria for the overview of reviews. If supplemental primary studies were included, this should be stated with a rationale.	6
	5b	Specify the definition of “systematic review” as used in the inclusion criteria for the overview ofreviews.	NO
Information sources	6	Specify all databases, registers, websites, organizations, reference lists, and other sources searched or consulted to identify systematic reviews and supplemental primary studies (if included). Specify the date when each source was last searched or consulted.	5-6
Search strategy	7	Present the full search strategies for all databases, registers, and websites, such that they could be reproduced. Describe any search filters and limits applied.	Suppl File 4
Selection process	8a	Describe the methods used to decide whether a systematic review or supplemental primary study (if included) met the inclusion criteria of the overview of reviews.	6-7
	8b	Describe how overlap in the populations, interventions, comparators, and/or outcomes of systematic reviews was identified and managed during study selection.	8-9
Data collection process	9a	Describe the methods used to collect data from reports.	8
	9b	If applicable, describe the methods used to identify and manage primary study overlap at the level of the comparison and outcome during data collection. For each outcome, specify the method used to illustrate and/or quantify the degree of primary study overlap across systematic reviews.	8-9
	9c	If applicable, specify the methods used to manage discrepant data across systematic reviews during data collection.	NO
Data items	10	List and define all variables and outcomes for which data were sought. Describe any assumptions made and/or measures taken to identify and clarify missing or unclear information.	6
Risk of bias assessment	11a	Describe the methods used to <u>*assess*</u> risk of bias or methodological quality of the included systematic reviews.	7-8
	11b	Describe the methods used to <u>*collect*</u> data on (from the systematic reviews) and/or <u>*assess*</u> the risk of bias of the primary studies included in the systematic reviews. Provide a justification for instances where flawed, incomplete, or missing assessments are identified but not re-assessed.	NO
	11c	Describe the methods used to <u>*assess*</u> the risk of bias of supplemental primary studies (if included).	Not applicable
Synthesis methods	12a	Describe the methods used to summarize or synthesize results and provide a rationale for the choice(s).	8 No rationale but justification was provided
	12b	Describe any methods used to explore possible causes of heterogeneity among results.	Not applicable
	12c	Describe any sensitivity analyses conducted to assess the robustness of the synthesized results.	Not applicable
Reporting bias assessment	13	Describe the methods used to <u>*collect*</u> data on (from the systematic reviews) and/or <u>*assess*</u> the risk of bias due to missing results in a summary or synthesis (arising from reporting biases at the levels of the systematic reviews, primary studies, and supplemental primary studies, if included).	NO
Certainty assessment	14	Describe the methods used to <u>*collect*</u> data on (from the systematic reviews) and/or <u>*assess*</u> certainty (or confidence) in the body of evidence for an outcome.	Not applicable
**RESULTS**			
Systematic review and supplemental	15a	Describe the results of the search and selection process, including the number of records screened, assessed for eligibility, and included in the overview of reviews, ideally with a flow diagram.	9 and Figure 1

Primary study selection	15b	Provide a list of studies that might appear to meet the inclusion criteria but were excluded, with the main reason for exclusion.	Suppl File 8

**Section** Topic	#	**Item**	**Location reported**
Characteristics of systematic reviews and supplemental primary studies	16	Cite each included systematic review and supplemental primary study (if included) and present its characteristics.	9 and Table 1
Primary study overlap	17	Describe the extent of primary study overlap across the included systematic reviews.	10 and Suppl Files 5-7
Risk of bias in systematic reviews, primary studies, and supplemental primary studies	18a	Present assessments of risk of bias or methodological quality for each included systematic review.	9 and Table 2
	18b	Present assessments (*collected* from systematic reviews or *assessed* anew) of the risk of bias of the primary studies included in the systematic reviews.	NO
	18c	Present assessments of the risk of bias of supplemental primary studies (if included).	Not applicable
Summary or synthesis of results	19a	For all outcomes, summarize the evidence from the systematic reviews and supplemental primary studies (if included). If meta-analyses were done, present for each the summary estimate and its precision and measures of statistical heterogeneity. If comparing groups, describe the direction of the effect.	10-11
	19b	If meta-analyses were done, present results of all investigations of possible causes of heterogeneity.	Not applicable
	19c	If meta-analyses were done, present results of all sensitivity analyses conducted to assess the robustness of synthesized results.	Not applicable
Reporting biases	20	Present assessments (*collected* from systematic reviews and/or *assessed* anew) of the risk of bias due to missing primary studies, analyses, or results in a summary or synthesis (arising from reporting biases at the levels of the systematic reviews, primary studies, and supplemental primary studies, if included) for each summary or synthesis assessed.	NO
Certainty of evidence	21	Present assessments (*collected* or *assessed* anew) of certainty (or confidence) in the body of evidence for each outcome.	Not applicable
**DISCUSSION**			
Discussion	22a	Summarize the main findings, including any discrepancies in findings across the included systematic reviews and supplemental primary studies (if included).	11
	22b	Provide a general interpretation of the results in the context of other evidence.	NO
	22c	Discuss any limitations of the evidence from systematic reviews, their primary studies, and supplemental primary studies (if included) included in the overview of reviews. Discuss any limitations of the overview of reviews methods used.	13
	22d	Discuss implications for practice, policy, and future research (both systematic reviews and primary research). Consider the relevance of the findings to the end users of the overview of reviews, e.g., healthcare providers, policymakers, patients, among others.	11-13
**OTHER INFORMATION**			
Registration and protocol	23a	Provide registration information for the overview of reviews, including register name and registration number, or state that the overview of reviews was not registered.	5 and abstract
	23b	Indicate where the overview of reviews protocol can be accessed, or state that a protocol was not prepared.	5 and abstract
	23c	Describe and explain any amendments to information provided at registration or in the protocol. Indicate the stage of the overview of reviews at which amendments were made.	Supplementary file 3
Support	24	Describe sources of financial or non-financial support for the overview of reviews, and the role of the funders or sponsors in the overview of reviews.	Title page and abstract
Competing interests	25	Declare any competing interests of the overview of reviews' authors.	Title page
Author information	26a	Provide contact information for the corresponding authors.	Title page
	26b	Describe the contributions of individual authors and identify the guarantor of the overview of reviews.	Title page
Availability of data and other materials	27	Report which of the following are available, where they can be found, and under which conditions they may be accessed: template data collection forms; data collected from included systematic reviews and supplemental primary studies; analytic code; any other materials used in the overview of reviews.	Not applicable
