# Supplementary material for: Improving Methodological Quality in Meta-Analyses of Athlete Pain Interventions: An Overview of Systematic Reviews
Source: Healthcare (Basel). 2025 Oct 2;13(19):2508. doi: 10.3390/healthcare13192508 (PMC12524677; doi:10.3390/healthcare13192508)
Supplement: Supplementary file 1 [file healthcare-13-02508-s001.zip › Suppl File 1 Search strategies.pdf]

Supplementary file 1. Search Strategies**.**

CINAHL (date 20/02/2025)

AB (athlete* OR player*) AND AB (pain) AND TI (systematic-review OR meta-analysis OR metaanalysis OR meta-analyses OR metaanalyses OR meta-review OR meta-analytic-review OR overview-of-systematic OR overview-of-reviews OR umbrella-review OR scoping-review)

Search modes - Proximity.

Search filters

Publication type: theses and dissertations were not considered.

Language of publication: no restrictions were imposed.

Studies retrieved = 131

Embase (date 20/02/2025)

(athlete*:ab,ti OR player*:ab,ti) AND (pain:ab,ti) AND (systematic-review:ti OR meta-analysis:ti OR metaanalysis:ti OR meta-analyses:ti OR metaanalyses:ti OR meta-review:ti OR meta-analytic-review:ti OR overview-of-systematic:ti OR overview-of-reviews:ti OR umbrella-review:ti OR scoping-review:ti)

Search filters

Publication type: conference abstracts were not considered in search strategies.

Language of publication: no restrictions were imposed.

Studies retrieved = 280

Epistemonikos (date 20/02/2025)

(title:((athlete* OR player*)) OR abstract:((athlete* OR player*))) AND (title:(pain) OR abstract:(pain)) AND title:((systematic-review OR meta-analysis OR metaanalysis OR

meta-analyses OR metaanalyses OR meta-review OR meta-analytic-review OR overview-of-systematic OR overview-of-reviews OR umbrella-review OR scoping-review))

Search filters

Publication type: no restrictions were imposed.

Language of publication: no restrictions were imposed.

Studies retrieved = 84

PubMed (date 20/02/2025)

(athlete* [tiab] OR player* [tiab]) AND (pain [tiab]) AND (systematic-review [title] OR meta-analysis [title] OR metaanalysis [title] OR meta-analyses [title] OR metaanalyses [title] OR meta-review [title] OR meta-analytic-review [title] OR overview-of-systematic [title] OR overview-of-reviews [title] OR umbrella-review [title] OR scoping review [title])

Search filters

Publication type: no restrictions were imposed.

Language of publication: no restrictions were imposed.

Studies retrieved = 310

Scopus (date 20/02/2025)

TITLE-ABS-KEY (athlete* OR player*) AND TITLE-ABS-KEY (pain) AND TITLE (systematic-review OR meta-analysis OR metaanalysis OR meta-analyses OR metaanalyses OR meta-review OR meta-analytic-review OR overview-of-systematic OR overview-of-reviews OR umbrella-review OR scoping-review)

Search filters

Publication type: no restrictions were imposed.

Language of publication: no restrictions were imposed.

Studies retrieved = 439

SPORTDiscus (date 20/02/2025)

AB (athlete* OR player*) AND AB (pain) AND TI (systematic-review OR meta-analysis OR metaanalysis OR meta-analyses OR metaanalyses OR meta-review OR meta-analytic-review OR overview-of-systematic OR overview-of-reviews OR umbrella-review OR scoping-review)

Search modes - Proximity.

Search filters

Publication type: no restrictions were imposed.

Language of publication: no restrictions were imposed.

Studies retrieved = 127

The Cochrane Library (date 20/02/2025)

ID	Search	Hits
#1	(athlete*):ti,ab,kw	10105
#2	(player*):ti,ab,kw	5887
#3	(pain):ti,ab,kw 260535	
#4	(#1 OR #2) AND (#3)	1729

Search filters

Type of document: Cochrane reviews and Cochrane protocols.

Language of publication: no restrictions were imposed.

Studies retrieved = 12 (12 Cochrane reviews and 0 Cochrane Protocols).
