# Supplementary material for: Improving Methodological Quality in Meta-Analyses of Athlete Pain Interventions: An Overview of Systematic Reviews
Source: Healthcare (Basel). 2025 Oct 2;13(19):2508. doi: 10.3390/healthcare13192508 (PMC12524677; doi:10.3390/healthcare13192508)
Supplement: Supplementary file 1 [file healthcare-13-02508-s001.zip › Suppl File 2 Excluded studies list.pdf]

Supplementary file 2. List of excluded studies and reasons after analysis at full text.

No analysis of interest: 1
No meta-analysis: 86
No meta-analysis of interest: 31
No outcome of interest: 6
No population of interest: 4
No research design of interest: 23
No full text available: 5

	**Excluded reviews**	**Reason**
1.	Afonso J, Claudino JG, Fonseca H, Moreira-Gonçalves D, Ferreira V, Almeida JM, Clemente FM, Ramirez-Campillo R. Stretching for Recovery from Groin Pain or Injury in Athletes: A Critical and Systematic Review. J Funct Morphol Kinesiol. 2021 Aug 30;6(3):73. doi: 10.3390/jfmk6030073.	No meta-analysis.
2.	Aldeeb M, Aminake GN, Khalil IA, Hayton M, Ksantini OEK, Hagert E. Isolated Trapezoid Fracture in Adolescent Goalkeepers: A Scoping Review of the Literature and a Report of Two Cases. J Hand Surg Glob Online. 2023 Oct 31;6(1):46-52. doi: 10.1016/j.jhsg.2023.09.001.	No meta-analysis.
3.	Alizadeh et al. The Effect of Prevention and Management Protocols on Low Back Pain in Athletes: A Systematic Review. Physical Treatments. 2022;12(4):233-248.	No meta-analysis.
4.	Almeida MO, Silva BN, Andriolo RB, Atallah AN, Peccin MS. Conservative interventions for treating exercise-related musculotendinous, ligamentous and osseous groin pain. Cochrane Database Syst Rev. 2013 Jun 6;2013(6):CD009565. doi: 10.1002/14651858.CD009565.pub2.	No meta-analysis of interest.<br>Note: Meta-analysis did not include two studies or more.
5.	Alvandi BA, Dayton SR, Hartwell MJ, Gerlach EB, Swiatek PR, Carney JJ, Tjong VK. Outcomes in Pediatric Hip FAI Surgery: a Scoping Review. Curr Rev Musculoskelet Med. 2022 Oct;15(5):362-368. doi: 10.1007/s12178-022-09771-6.	No research design of interest.
6.	Annin S, Lall AC, Yelton MJ, Shapira J, Rosinsky PJ, Meghpara MB, Maldonado DR, Ankem H, Domb BG. Patient-Reported Outcomes in Athletes Following Hip Arthroscopy for Femoroacetabular Impingement With Subanalysis on Return to Sport and Performance Level: A Systematic Review. Arthroscopy. 2021 Aug;37(8):2657-2676. doi: 10.1016/j.arthro.2021.03.064.	No meta-analysis.

7.	Apostolopoulos N, Metsios GS, Flouris AD, Koutedakis Y, Wyon MA. The relevance of stretch intensity and position-a systematic review. Front Psychol. 2015 Aug 18;6:1128. doi: 10.3389/fpsyg.2015.01128.	No meta-analysis.
8.	Benoit-Piau J, Benoit-Piau C, Gaudreault N, Morin M. Effect of Conservative Interventions for Musculoskeletal Disorders in Preprofessional and Professional Dancers: A Systematic Review. Int J Sports Phys Ther. 2023 Apr 1;18(2):328-337. doi: 10.26603/001c.73793.	No meta-analysis.
9.	Bethell MA, Hurley ET, Rowe D, Crook BS, Cabell G, Klifto CS, Lau BC, Dickens JF, Taylor DC. Type V superior labrum anterior to posterior repair: a systematic review. J Shoulder Elbow Surg. 2024 Aug;33(8):e443-e450. doi: 10.1016/j.jse.2024.01.054.	No population of interest.<br>Note: The authors did not specify in the objective or eligibility criteria if all the participants were athletes.
10.	Bieleke et al. If-Then Planning in Sports: A Scoping Review. Zeitschrift fur Sportpsychologie. 2021;28(3):109-120.	No research design of interest.
11.	Bisciotti GN, Chamari K, Cena E, Garcia GR, Vuckovic Z, Bisciotti A, Bisciotti A, Zini R, Corsini A, Volpi P. The conservative treatment of longstanding adductor-related groin pain syndrome: a critical and systematic review. Biol Sport. 2021 Mar;38(1):45-63. doi: 10.5114/biolsport.2020.97669.	No meta-analysis.
12.	Bolia IK, Gammons P, Scholten DJ, Weber AE, Waterman BR. Operative Versus Nonoperative Management of Distal Iliotibial Band Syndrome-Where Do We Stand? A Systematic Review. Arthrosc Sports Med Rehabil. 2020 Jun 10;2(4):e399-e415. doi: 10.1016/j.asmr.2020.04.001.	No meta-analysis.
13.	Bozzo A, Oitment C, Thornley P, Yan J, Habib A, Hoppe DJ, Athwal GS, Ayeni OR. Humeral Avulsion of the Glenohumeral Ligament: Indications for Surgical Treatment and Outcomes-A Systematic Review. Orthop J Sports Med. 2017 Aug 14;5(8):2325967117723329. doi: 10.1177/2325967117723329.	No meta-analysis.
14.	Brito R, Cruz P, Costa D, Afonso S, Barros P. Nonsurgical Interventions for the Management of Long-Standing Groin Pain in Athletes: A Systematic Review of Randomized Controlled Trials. Cureus. 2023 Jun 8;15(6):e40149. doi: 10.7759/cureus.40149.	No meta-analysis.

15.	Burton I. Interventions for prevention and in-season management of patellar tendinopathy in athletes: A scoping review. Phys Ther Sport. 2022 May;55:80-89. doi: 10.1016/j.ptsp.2022.03.002.	No research design of interest.
16.	Bridgett R, Klose P, Duffield R, Mydock S, Lauche R. Effects of Cupping Therapy in Amateur and Professional Athletes: Systematic Review of Randomized Controlled Trials. J Altern Complement Med. 2018 Mar;24(3):208-219. doi: 10.1089/acm.2017.0191.	No meta-analysis.
17.	Brown CK, Southerst D, Côté P, Shearer HM, Randhawa K, Wong JJ, Yu H, Varatharajan S, Sutton D, Stern PJ, D'Angelo K, Dion S, Cox J, Goldgrub R, Stupar M, Carroll LJ, Taylor-Vaisey A. The Effectiveness of Exercise on Recovery and Clinical Outcomes in Patients With Soft Tissue Injuries of the Hip, Thigh, or Knee: A Systematic Review by the Ontario Protocol for Traffic Injury Management (OPTIMa) Collaboration. J Manipulative Physiol Ther. 2016 Feb;39(2):110-120.e1. doi: 10.1016/j.jmpt.2016.01.003.	No meta-analysis.
18.	Chaabna K, Jithesh A, Cheema J, Aboughanem J, Mamtani R. Western Medical Acupuncture Perception and Use for Pain Management Among Athletes: A Systematic Review. J Pain Res. 2024 Jan 26;17:357-366. doi: 10.2147/JPR.S441869.	No meta-analysis.
19.	Charlton PC, Drew MK, Mentiplay BF, Grimaldi A, Clark RA. Exercise Interventions for the Prevention and Treatment of Groin Pain and Injury in Athletes: A Critical and Systematic Review. Sports Med. 2017 Oct;47(10):2011-2026. doi: 10.1007/s40279-017-0742-y.	No meta-analysis.
20.	Choi H, McCartney M, Best TM. Treatment of osteitis pubis and osteomyelitis of the pubic symphysis in athletes: a systematic review. Br J Sports Med. 2011 Jan;45(1):57-64. doi: 10.1136/bjsm.2008.050989.	No meta-analysis.
21.	Chona DV, Minetos PD, LaPrade CM, Cinque ME, Abrams GD, Sherman SL, Safran MR. Hip Dislocation and Subluxation in Athletes: A Systematic Review. Am J Sports Med. 2022 Aug;50(10):2834-2841. doi: 10.1177/03635465211036104.	No analysis of interest.<br>Note: The authors did not report in their analysis standardized mean differences or mean differences for pain.

22.	Cognetti DJ, Sheean AJ, Arner JW, Wilkerson D, Bradley JP. Surgical Management of Patellar Tendinopathy Results in Improved Outcomes and High Rates of Return to Sport: A Systematic Review. J Knee Surg. 2023 Sep;36(11):1171-1190. doi: 10.1055/s-0042-1757701.	No full text available.<br><br>Note: We were unable to contact the authors because we did not find in the journal where the article was published nor the corresponding author's email address on the website where the article was published.
23.	Cope T, Wechter S, Stucky M, Thomas C, Wilhelm M. THE IMPACT OF LUMBOPELVIC CONTROL ON OVERHEAD PERFORMANCE AND SHOULDER INJURY IN OVERHEAD ATHLETES: A SYSTEMATIC REVIEW. Int J Sports Phys Ther. 2019 Jul;14(4):500-513.	No meta-analysis.
24.	Córdoba LL, Rodrigues MC, Corrêa de Freitas R, Neto HP, Serafim Bonvino MA, Rossi MF, Mazzei LG. Physiotherapeutic approach to the preoperative period for the anterior cruciate ligament reconstruction: A systematic review. J Bodyw Mov Ther. 2023 Jan;33:88-94. doi: 10.1016/j.jbmt.2022.09.001.	No meta-analysis.
25.	Cornu C, Grange C, Regalin A, Munier J, Ounissi S, Reynaud N, Kassai-Koupai B, Sallet P, Nony P. Effect of Non-Steroidal Anti-Inflammatory Drugs on Sport Performance Indices in Healthy People: a Meta-Analysis of Randomized Controlled Trials. Sports Med Open. 2020 Apr 28;6(1):20. doi: 10.1186/s40798-020-00247-w.	No meta-analysis of interest.<br><br>Note: The authors did not specify in data analysis that subgroups including only athletes were performed.
26.	Costello JT, Baker PR, Minett GM, Bieuzen F, Stewart IB, Bleakley C. Whole-body cryotherapy (extreme cold air exposure) for preventing and treating	No meta-analysis of interest.

	muscle soreness after exercise in adults. Cochrane Database Syst Rev. 2015 Sep 18;2015(9):CD010789. doi: 10.1002/14651858.CD010789.pub2.	Note: No specific meta-analyses focused on athletes were performed.
27.	Crepaldi et al. Nutrological aspects of polyphenols and gut microbiota in sports performance: a systematic review. International Journal of Nutrology. 2024;17(1).	No meta-analysis.
28.	Culvenor AG, van Middelkoop M, Macri EM, Crossley KM. Is patellofemoral pain preventable? A systematic review and meta-analysis of randomised controlled trials. Br J Sports Med. 2020 Oct 28:bjsports-2020-102973. doi: 10.1136/bjsports-2020-102973.	No outcome of interest.<br>Note: The authors evaluated the risk of patellofemoral pain.
29.	Cusano, A., Ment, A.J., Hao, K.A., Saleet, J., Nian, P.P., Curry, E.J., Groot, M., Novikov, D., Abdul-Rassoul, H., Parisien, R.L., Owens, B.D., & Li, X. (2024). Arthroscopic Repair for Posterior Shoulder Instability is Associated with Favorable Outcomes and High Return to Sport or Work: A Systematic Review and Meta-Analysis. Arthroscopy, Sports Medicine, and Rehabilitation.	No outcome of interest.
30.	D'Ambrosi R, Meena A, Raj A, Ursino N, Mangiavini L, Herbort M, Fink C. In elite athletes with meniscal injuries, always repair the lateral, think about the medial! A systematic review. Knee Surg Sports Traumatol Arthrosc. 2023 Jun;31(6):2500-2510. doi: 10.1007/s00167-022-07208-8.	No meta-analysis.
31.	Daniel Vasile PR, Patricia ML, Marta MS, Laura E. Evaluation of curcumin intake in reducing exercise-induced muscle damage in athletes: a systematic review. J Int Soc Sports Nutr. 2024 Dec;21(1):2434217. doi: 10.1080/15502783.2024.2434217.	No meta-analysis.
32.	Dakić M, Toskić L, Ilić V, Đurić S, Dopsaj M, Šimenko J. The Effects of Massage Therapy on Sport and Exercise Performance: A Systematic Review. Sports (Basel). 2023 May 29;11(6):110. doi: 10.3390/sports11060110.	No meta-analysis.
33.	de Castro Fernendes et al. Comparative analysis of treatment strategies for groin injuries in athletes: Effects on successful recovery and recurrence of sports conditions – A systematic review. Apunts Sports Medicine Volume. 2025;60(227):100481.	No meta-analysis.

34.	de Oliveira et al. Effect of single and multiple sessions of self-myofascial release: systematic review. Revista Brasileira de Medicina do Esporte. 2022;28(4):358-67.	No meta-analysis.
35.	de Sa D, Hölmich P, Phillips M, Heaven S, Simunovic N, Philippon MJ, Ayeni OR. Athletic groin pain: a systematic review of surgical diagnoses, investigations and treatment. Br J Sports Med. 2016 Oct;50(19):1181-6. doi: 10.1136/bjsports-2015-095137.	No meta-analysis.
36.	de Sire A, Marotta N, Prestifilippo E, Parente A, Lopresti E, Drago Ferrante V, Sgro M, Lippi L, Invernizzi M, Ammendolia A. Efficacy of platelet-rich plasma injection for pain relief in injured athletes: a systematic review of randomized controlled trials. J Sports Med Phys Fitness. 2025 Jan 13. doi: 10.23736/S0022-4707.24.16572-3.	No meta-analysis.
37.	de Vos RJ, Windt J, Weir A. Strong evidence against platelet-rich plasma injections for chronic lateral epicondylar tendinopathy: a systematic review. Br J Sports Med. 2014 Jun;48(12):952-6. doi: 10.1136/bjsports-2013-093281.	No meta-analysis.
38.	Dehghan F, Fouladi R, Martin J. Kinesio taping in sports: A scoping review. J Bodyw Mov Ther. 2024 Oct;40:1213-1223. doi: 10.1016/j.jbmt.2023.05.008.	No research design of interest.
39.	Demeco A, de Sire A, Salerno A, Marotta N, Palermi S, Frizziero A, Costantino C. Dry Needling in Overhead Athletes with Myofascial Shoulder Pain: A Systematic Review. Sports (Basel). 2024 Jun 5;12(6):156. doi: 10.3390/sports12060156.	No meta-analysis.
40.	Di Dio M, Calella P, Pelullo CP, Liguori F, Di Onofrio V, Gallè F, Liguori G. Effects of Probiotic Supplementation on Sports Performance and Performance-Related Features in Athletes: A Systematic Review. Int J Environ Res Public Health. 2023 Jan 26;20(3):2226. doi: 10.3390/ijerph20032226.	No meta-analysis.
41.	Duarte Franca et al. Effect of myofascial release techniques on internal biomechanics and their resultant application to sports: A systematic review. Journal of Bodywork and Movement Therapies. 2024;40:525-533.	No meta-analysis.
42.	Elmeligie et al. Effectiveness of cupping therapy for musculoskeletal pain: an umbrella review. Human Movement. 2024;25(4):28-43.	No research design of interest.

43.	Evans S. Sacroiliac Joint Dysfunction in Endurance Runners Using Wearable Technology as a Clinical Monitoring Tool: Systematic Review. JMIR Biomed Eng. 2024 May 20;9:e46067. doi: 10.2196/46067.	No meta-analysis.
44.	Farrell SG, Hatem M, Bharam S. Acute Adductor Muscle Injury: A Systematic Review on Diagnostic Imaging, Treatment, and Prevention. Am J Sports Med. 2023 Nov;51(13):3591-3603. doi: 10.1177/03635465221140923.	No meta-analysis.
45.	Fernandes et al. Does Quercetin Supplementation Promote Biological Changes and Performance in Athletes? A Systematic Review. Journal of Biological Regulators and Homeostatic Agents. 2024;38(7):5371-5381.	No meta-analysis.
46.	Ferreira Santana, S.D., Gava, V., Alves de Oliveira, V.M., Alves dos Santos, G., Soares Lima, T.A., & Kamonseki, D.H. (2025). Does manual therapy improve pain, disability, and glenohumeral motion in overhead athletes? Systematic review and meta-analysis. International Journal of Osteopathic Medicine.	No meta-analysis of interest.<br>Note: Only one study was included evaluating pain intensity.
47.	Forogh B, Ghaseminejad Raeini A, Jebeli Fard R, Mirghaderi P, Nakhostin-Ansari A, Nakhostin-Ansari N, Bahari H, Hoveidaei AH. Efficacy of trigger point dry needling on pain and function of the hip joint: a systematic review of randomized clinical trials. Acupunct Med. 2024 Apr;42(2):63-75. doi: 10.1177/09645284231207870.	No meta-analysis.
48.	Francisco Pereira et al. Efecto de la meditación mindfulness en el deporte de alto rendimiento: una revisión del alcance (Effect of Mindfulness Meditation on High-Performance Sports: A Scoping Review). Retos: Nuevas Perspectivas de Educación Física, Deporte y Recreación. 2024;57:536-560.	No meta-analysis.
49.	Frantz TL, Shacklett AG, Martin AS, Barlow JD, Jones GL, Neviaser AS, Cvetanovich GL. Biceps Tenodesis for Superior Labrum Anterior-Posterior Tear in the Overhead Athlete: A Systematic Review. Am J Sports Med. 2021 Feb;49(2):522-528. doi: 10.1177/0363546520921177.	No meta-analysis.
50.	Furlan MR, Machado E, do Nascimento Petter G, Barbosa IM, Geremia JM, Glänzel MH. Self-Massage Acute Effects on Pressure Pain Threshold, Muscular Electrical Activity, and Muscle Force Production: A Systematic Review and Meta-Analysis. J Strength Cond Res. 2024 Mar 1;38(3):620-635. doi: 10.1519/JSC.0000000000004721.	No outcome of interest.<br>Note: Pressure pain threshold.

51.	Gennarelli SM, Brown SM, Mulcahey MK. Psychosocial interventions help facilitate recovery following musculoskeletal sports injuries: a systematic review. Phys Sportsmed. 2020 Nov;48(4):370-377. doi: 10.1080/00913847.2020.1744486.	No meta-analysis.
52.	Gombera MM, Sekiya JK. Rotator cuff tear and glenohumeral instability : a systematic review. Clin Orthop Relat Res. 2014 Aug;472(8):2448-56. doi: 10.1007/s11999-013-3290-2. Erratum in: Clin Orthop Relat Res. 2015 Feb;473(2):751. Gomberawalla, M Mustafa [corrected to Gombera, Mufaddal Mustafa].	No meta-analysis.
53.	Gouveia K, Kay J, Memon M, Simunovic N, Bedi A, Ayeni OR. Return to Sport After Surgical Management of Posterior Shoulder Instability: A Systematic Review and Meta-analysis. Am J Sports Med. 2022 Mar;50(3):845-857. doi: 10.1177/03635465211011161.	No meta-analysis of interest.
54.	Gracitelli GC, Moraes VY, Franciozi CE, Luzo MV, Belloti JC. Surgical interventions (microfracture, drilling, mosaicplasty, and allograft transplantation) for treating isolated cartilage defects of the knee in adults. Cochrane Database Syst Rev. 2016 Sep 3;9(9):CD010675. doi: 10.1002/14651858.CD010675.pub2.	No meta-analysis of interest.<br>Note: Only one study evaluated pain.
55.	Grassi A, Napoli F, Romandini I, Samuelsson K, Zaffagnini S, Candrian C, Filardo G. Is Platelet-Rich Plasma (PRP) Effective in the Treatment of Acute Muscle Injuries? A Systematic Review and Meta-Analysis. Sports Med. 2018 Apr;48(4):971-989. doi: 10.1007/s40279-018-0860-1.	No meta-analysis of interest.
56.	Gultekin S, Chaker Jomaa M, Jenkin R, Orchard JW. Use and Outcome of Local Anesthetic Painkilling Injections in Athletes: A Systematic Review. Clin J Sport Med. 2021 Jan;31(1):78-85. doi: 10.1097/JSM.0000000000000716.	No meta-analysis.
57.	Hameed I, Farooq N, Haq A, Aimen I, Shanley J. Role of strengthening exercises in management and prevention of overuse sports injuries of lower extremity: a systematic review. J Sports Med Phys Fitness. 2024 Aug;64(8):807-815. doi: 10.23736/S0022-4707.23.15470-3.	No full text available.<br>Note: This article was requested by the authors, but we have not received any response.

58.	Harle CA, Danielson EC, Derman W, Stuart M, Dvorak J, Smith L, Hainline B. Analgesic Management of Pain in Elite Athletes: A Systematic Review. Clin J Sport Med. 2018 Sep;28(5):417-426. doi: 10.1097/JSM.0000000000000604.	No meta-analysis.
59.	Hatem M, Martin RL, Bharam S. Surgical Outcomes of Inguinal-, Pubic-, and Adductor-Related Chronic Pain in Athletes: A Systematic Review Based on Surgical Technique. Orthop J Sports Med. 2021 Sep 13;9(9):23259671211023116. doi: 10.1177/23259671211023116.	No meta-analysis of interest.
60.	Herman K, Barton C, Malliaras P, Morrissey D. The effectiveness of neuromuscular warm-up strategies, that require no additional equipment, for preventing lower limb injuries during sports participation: a systematic review. BMC Med. 2012 Jul 19;10:75. doi: 10.1186/1741-7015-10-75.	No meta-analysis.
61.	Hickey et al. The Long-Term Effects of Eccentric Exercise vs. Extracorporeal Shockwave Therapy in Athletes Aged 18-50 with Patellar Tendinopathy: A Meta-Analysis and Systematic Review. Annals of Physiotherapy & Occupational Therapy. 2019;2(2):1-16.	No research design of interest.
62.	Hurley ET, Jamal MS, Ali ZS, Montgomery C, Pauzenberger L, Mullett H. Long-term outcomes of the Latarjet procedure for anterior shoulder instability: a systematic review of studies at 10-year follow-up. J Shoulder Elbow Surg. 2019 Feb;28(2):e33-e39. doi: 10.1016/j.jse.2018.08.028.	No meta-analysis of interest.
63.	Hurley ET, Baker R, Danilkowicz RM, Levin JM, Klifto CS, Dickens JF, Taylor DC, Lau BC. Similar outcomes between biceps tenodesis and SLAP repair for SLAP tears in younger patients - A meta-analysis. J ISAKOS. 2024 Feb;9(1):79-83. doi: 10.1016/j.jisako.2023.09.007.	No meta-analysis of interest.<br>Note: The authors did not specify in the objective or eligibility criteria that athletes were considered for pain.
64.	Ifabiyi M, Patel M, Cohen D, Simunovic N, Ayeni OR. Return-to-Sport Rates After Hip Arthroscopy for Femoroacetabular Impingement Syndrome in Flexibility Sports Athletes: A Systematic Review. Sports Health. 2024 Nov-Dec;16(6):982-990. doi: 10.1177/19417381231217503.	No meta-analysis of interest.
65.	Jankaew A, Chen JC, Chamnongkich S, Lin CF. Therapeutic Exercises and Modalities in Athletes With Acute Hamstring Injuries: A Systematic Review	No meta-analysis of interest.

	and Meta-analysis. Sports Health. 2023 Jul-Aug;15(4):497-511. doi: 10.1177/19417381221118085.	Note: Pain intensity was not meta-analyzed.
66.	Jansen JA, Mens JM, Backx FJ, Kolfschoten N, Stam HJ. Treatment of longstanding groin pain in athletes: a systematic review. Scand J Med Sci Sports. 2008 Jun;18(3):263-74. doi: 10.1111/j.1600-0838.2008.00790.x.	No meta-analysis.
67.	Jelsema TR, Tam AC, Moeller JL. Injectable Ketorolac and Corticosteroid Use in Athletes: A Systematic Review. Sports Health. 2020 Nov/Dec;12(6):521-527. doi: 10.1177/1941738120946008.	No meta-analysis.
68.	Jinnah et al. Cryotherapy duration is critical in short-term recovery of athletes: A systematic review. Journal of ISAKOS. 2019;4(3):131-136.	No meta-analysis.
69.	Jones L, Bailey SJ, Rowland SN, Alsharif N, Shannon OM, Clifford T. The Effect of Nitrate-Rich Beetroot Juice on Markers of Exercise-Induced Muscle Damage: A Systematic Review and Meta-Analysis of Human Intervention Trials. J Diet Suppl. 2022;19(6):749-771. doi: 10.1080/19390211.2021.1939472.	No meta-analysis of interest.<br>Note: The authors did not specify in the objective or eligibility criteria that athletes were specifically analyzed.
70.	Karasuyama M, Oike T, Okamatsu S, Kawakami J. Shoulder pain in wheelchair basketball athletes: A scoping review. J Spinal Cord Med. 2023 Sep;46(5):753-759. doi: 10.1080/10790268.2022.2038050.	No research design of interest.
71.	Kasik C, Saper MG. Variability of Outcome Reporting Following Arthroscopic Bankart Repair in Adolescent Athletes: A Systematic Review. Arthroscopy. 2018 Apr;34(4):1288-1294. doi: 10.1016/j.arthro.2017.10.041.	No meta-analysis.
72.	Kearney RS, Parsons N, Metcalfe D, Costa ML. Injection therapies for Achilles tendinopathy. Cochrane Database Syst Rev. 2015 May 26;2015(5):CD010960. doi: 10.1002/14651858.CD010960.pub2.	No meta-analysis of interest.<br>Note: Meta-analysis evaluating pain did not separate athletes and non-

		athletic populations.
73.	Khatri M, Naughton RJ, Clifford T, Harper LD, Corr L. The effects of collagen peptide supplementation on body composition, collagen synthesis, and recovery from joint injury and exercise: a systematic review. Amino Acids. 2021 Oct;53(10):1493-1506. doi: 10.1007/s00726-021-03072-x.	No meta-analysis.
74.	King E, Ward J, Small L, Falvey E, Franklyn-Miller A. Athletic groin pain: a systematic review and meta-analysis of surgical versus physical therapy rehabilitation outcomes. Br J Sports Med. 2015 Nov;49(22):1447-51. doi: 10.1136/bjsports-2014-093715.	No outcome of interest.
75.	Kler A, Sekhon N, Antoniou GA, Satyadas T. Totally extra-peritoneal repair versus trans-abdominal pre-peritoneal repair for the laparoscopic surgical management of sportsman's hernia: A systematic review and meta-analysis. Surg Endosc. 2021 Oct;35(10):5399-5413. doi: 10.1007/s00464-021-08554-3.	No meta-analysis of interest.
76.	Koerber SN, Wager SG, Zynda AJ, Santa Barbara MT. Scoping Review: Reducing Musculoskeletal Injury Risk Factors for Adaptive Sport Athletes Through Prevention Programs. Am J Phys Med Rehabil. 2024 Nov 1;103(11):1045-1050. doi: 10.1097/PHM.0000000000002490.	No research design of interest.
77.	Konrad A, Močnik R, Nakamura M. Effects of Tissue Flossing on the Healthy and Impaired Musculoskeletal System: A Scoping Review. Front Physiol. 2021 May 21;12:666129. doi: 10.3389/fphys.2021.666129.	No research design of interest.
78.	Kramer A, Sinclair J, Sharpe L, Sarris J. Chronic cannabis consumption and physical exercise performance in healthy adults: a systematic review. J Cannabis Res. 2020 Oct 7;2(1):34. doi: 10.1186/s42238-020-00037-x.	No meta-analysis.
79.	Križaj L, Kozinc Ž, Löfler S, Šarabon N. The chronic effects of eccentric exercise interventions in different populations: an umbrella review. Eur J Transl Myol. 2022 Oct 21;32(4):10876. doi: 10.4081/ejtm.2022.10876.	No research design of interest.
80.	Kunene SH, Taukobong NP, Ramklass S. Rehabilitation approaches to anterior knee pain among runners: A scoping review. S Afr J Physiother. 2020 Jan 27;76(1):1342. doi: 10.4102/sajp.v76i1.1342.	No research design of interest.
81.	Kuźdżał A, Trybulski R, Muracki J, Klich S, Clemente FM, Kawczyński A. Dry Needling in Sports and Sport Recovery: A Systematic Review with an	No meta-analysis.

	Evidence Gap Map. Sports Med. 2025 Feb 10. doi: 10.1007/s40279-025-02175-9.	
82.	Lee JW, Lee JH, Kim SY. Use of Acupuncture for the Treatment of Sports-Related Injuries in Athletes: A Systematic Review of Case Reports. Int J Environ Res Public Health. 2020 Nov 6;17(21):8226. doi: 10.3390/ijerph17218226.	No meta-analysis.
83.	Lewis T, Cook J. Fluoroquinolones and tendinopathy: a guide for athletes and sports clinicians and a systematic review of the literature. J Athl Train. 2014 May-Jun;49(3):422-7. doi: 10.4085/1062-6050-49.2.09.	No meta-analysis.
84.	Liddle AD, Rodríguez-Merchán EC. Platelet-Rich Plasma in the Treatment of Patellar Tendinopathy: A Systematic Review. Am J Sports Med. 2015 Oct;43(10):2583-90. doi: 10.1177/0363546514560726.	No meta-analysis.
85.	Liu, S., & Noh, Y. E. (2024). The effectiveness and applicability of mindfulness intervention in psychological adaptation after sports injury: a systematic review. Australian Journal of Psychology, 76(1). https://doi.org/10.1080/00049530.2024.2357627	No meta-analysis.
86.	Lorenzen J, Krämer R, Vogt PM, Knobloch K. Systematische Literaturanalyse über exzentrisches Training bei chronischer Patellatendinopathie: Gibt es einen Standard? [Systematic review about eccentric training in chronic patella tendinopathy]. Sportverletz Sportschaden. 2010 Dec;24(4):198-203. German. doi: 10.1055/s-0029-1245818. E	No full text available.<br><br>Note: We were unable to contact the authors.
87.	Lu X, Wang Y, Lu J, You Y, Zhang L, Zhu D, Yao F. Does vibration benefit delayed-onset muscle soreness?: a meta-analysis and systematic review. J Int Med Res. 2019 Jan;47(1):3-18. doi: 10.1177/0300060518814999.	No meta-analysis of interest.<br><br>Note: No specific meta-analyses focused on athletes were performed.
88.	Machotka Z, Kumar S, Perraton LG. A systematic review of the literature on the effectiveness of exercise therapy for groin pain in athletes. Sports Med Arthrosc Rehabil Ther Technol. 2009 Mar 31;1(1):5. doi: 10.1186/1758-2555-1-5.	No meta-analysis.

89.	Manjunath AK, Hurley ET, Jazrawi LM, Strauss EJ. Return to Play After Medial Patellofemoral Ligament Reconstruction: A Systematic Review. Am J Sports Med. 2021 Mar;49(4):1094-1100. doi: 10.1177/0363546520947044.	No meta-analysis.
90.	Masoudi A, Chemane N, Magida N, Useh U, Bello B. Effectiveness of self-management programmes for athletes with patellofemoral pain syndrome: A systematic review. S Afr J Sports Med. 2025 Feb 15;37(1):v37i1a18648. doi: 10.17159/2078-516X/2025/v37i1a18648.	No meta-analysis.
91.	Marco et al. Effectiveness of a Long-Term Tecar Therapy Treatment on Knee Pain: Building T.T.E.S.S.K an Evaluating Scale-A Systematic Review and Meta-Analysis. Systematic Reviews in Pharmacy. 2022;13(9):587-599.	No research design of interest.
92.	Marcon et al. The effect of branched-chain amino acids supplementation in physical exercise: A systematic review of human randomized controlled trials. Science & Sports. 2022;37(5–6):393-404.	No meta-analysis.
93.	Marriott KA, Hall M, Maciukiewicz JM, Almaw RD, Wiebenga EG, Ivanochko NK, Rinaldi D, Tung EV, Bennell KL, Maly MR. Are the Effects of Resistance Exercise on Pain and Function in Knee and Hip Osteoarthritis Dependent on Exercise Volume, Duration, and Adherence? A Systematic Review and Meta-Analysis. Arthritis Care Res (Hoboken). 2024 Jun;76(6):821-830. doi: 10.1002/acr.25313.	No population of interest.
94.	Mason DL, Dickens VA, Vail A. Rehabilitation for hamstring injuries. Cochrane Database Syst Rev. 2012 Dec 12;12:CD004575. doi: 10.1002/14651858.CD004575.pub3.	No outcome of interest.
95.	Matar RN, Shah NS, Gardner TJ, Grawe BM. Return to sport after surgical treatment for posterior shoulder instability: a systematic review. JSES Int. 2020 Sep 11;4(4):797-802. doi: 10.1016/j.jseint.2020.08.002.	No meta-analysis.
96.	Memon M, Kay J, Cadet ER, Shahsavar S, Simunovic N, Ayeni OR. Return to sport following arthroscopic Bankart repair: a systematic review. J Shoulder Elbow Surg. 2018 Jul;27(7):1342-1347. doi: 10.1016/j.jse.2018.02.044.	No meta-analysis of interest.
97.	Minhaj S, Afridi ZK, Rubab S, Qazi Z, Siddiqui M. Glenohumeral Internal Rotation Deficit and Risk of Upper Extremity Injury in Overhead Athletes: Systematic Review. Arch Phys Med Rehabil. 2025 Jan;106(1):91-97. doi: 10.1016/j.apmr.2024.05.027.	No meta-analysis.

98.	Mohamed AA, Zhang X, Jan Y-K. Evidence-based and adverse-effects analyses of cupping therapy in musculoskeletal and sports rehabilitation: a systematic and evidence-based review. J Back Musculoskelet Rehabil. 2023;36:3–19; doi: 10.3233/ BMR-210242.	No meta-analysis of interest.
99.	Mohd Tan A, Zahari Z, Bukry SA. Strengthening exercise and motor control among football players with ankle sprain: A scoping review. Med J Malaysia. 2024 Mar;79(Suppl 1):197-202.	No research design of interest.
100.	Momaya AM, Kwapisz A, Choate WS, Kissenberth MJ, Tolan SJ, Lonergan KT, Hawkins RJ, Tokish JM. Clinical outcomes of suprascapular nerve decompression: a systematic review. J Shoulder Elbow Surg. 2018 Jan;27(1):172-180. doi: 10.1016/j.jse.2017.09.025.	No meta-analysis.
101.	Montalvo AM, Cara EL, Myer GD. Effect of kinesiology taping on pain in individuals with musculoskeletal injuries: systematic review and meta-analysis. Phys Sportsmed. 2014 May;42(2):48-57. doi: 10.3810/psm.2014.05.2057.	No population of interest.
102.	Morath O, Beck M, Taeymans J, Hirschmüller A. Sclerotherapy and prolotherapy for chronic patellar tendinopathies - a promising therapy with limited available evidence, a systematic review. J Exp Orthop. 2020 Nov 9;7(1):89. doi: 10.1186/s40634-020-00303-0.	No meta-analysis.
103.	Moretti A, Palomba A, Paoletta M, Liguori S, Toro G, Iolascon G. Complex Regional Pain Syndrome in Athletes: Scoping Review. Medicina (Kaunas). 2021 Nov 17;57(11):1262. doi: 10.3390/medicina57111262.	No research design of interest.
104.	Morgan S, Janse van Vuuren EC, Coetzee FF. Causative factors and rehabilitation of patellar tendinopathy: A systematic review. S Afr J Physiother. 2016 Nov 29;72(1):338. doi: 10.4102/sajp.v72i1.338.	No meta-analysis.
105.	Morone G, Ghanbari Ghooshchy S, Pulcini C, Spangu E, Zoccolotti P, Martelli M, Spitoni GF, Russo V, Ciancarelli I, Paolucci S, et al. Motor Imagery and Sport Performance: A Systematic Review on the PETTLEP Model. Applied Sciences. 2022; 12(19):9753. https://doi.org/10.3390/app12199753	No meta-analysis.
106.	Morrissey CD, Knapik DM. Prevalence, Mechanisms, and Return to Sport After Isolated Popliteus Injuries in Athletes: A Systematic Review. Orthop J Sports Med. 2022 Feb 28;10(2):23259671211073617. doi: 10.1177/23259671211073617.	No meta-analysis.

107.	Mostafavifar M, Wertz J, Borchers J. A systematic review of the effectiveness of kinesio taping for musculoskeletal injury. Phys Sportsmed. 2012 Nov;40(4):33-40. doi: 10.3810/psm.2012.11.1986.	No meta-analysis.
108.	Multhaupt et al. The use of imagery in athletic injury rehabilitation. A systematic review. Deutsche Zeitschrift fur Sportmedizin. 2018;69(3):57-64.	No meta-analysis.
109.	Murray A, Cardinale M. Cold applications for recovery in adolescent athletes: a systematic review and meta analysis. Extrem Physiol Med. 2015 Oct 12;4:17. doi: 10.1186/s13728-015-0035-8.	No outcome of interest.
110.	Nazari G, Bobos P, Lu SZ, Reischl S, Sharma S, Le CY, Vader K, Held N, MacDermid JC. Effectiveness of instrument-assisted soft tissue mobilization for the management of upper body, lower body, and spinal conditions. An updated systematic review with meta-analyses. Disabil Rehabil. 2023 May;45(10):1608-1618. doi: 10.1080/09638288.2022.2070288.	No meta-analysis of interest.
111.	Nazari G, Bobos P, MacDermid JC, Birmingham T. The Effectiveness of Instrument-Assisted Soft Tissue Mobilization in Athletes, Participants Without Extremity or Spinal Conditions, and Individuals with Upper Extremity, Lower Extremity, and Spinal Conditions: A Systematic Review. Arch Phys Med Rehabil. 2019 Sep;100(9):1726-1751. doi: 10.1016/j.apmr.2019.01.017. E	No meta-analysis.
112.	Paajanen H, Montgomery A, Simon T, Sheen AJ. Systematic review: laparoscopic treatment of long-standing groin pain in athletes. Br J Sports Med. 2015 Jun;49(12):814-8. doi: 10.1136/bjsports-2014-094544.	No meta-analysis.
113.	Papalia R, Ciuffreda M, Albo E, De Andreis C, Diaz Balzani LA, Alifano AM, Fossati C, Macaluso A, Borzuola R, De Vincentis A, Denaro V. Return to Sport after Anatomic and Reverse Total Shoulder Arthroplasty in Elderly Patients: A Systematic Review and Meta-Analysis. J Clin Med. 2020 May 22;9(5):1576. doi: 10.3390/jcm9051576.	No meta-analysis of interest.
114.	Paulino Pereira NR, van der Linde JA, Alkaduhimi H, Longo UG, van den Bekerom MPJ. Are collision athletes at a higher risk of re-dislocation after an open Bristow-Latarjet procedure? A systematic review and meta-analysis. Shoulder Elbow. 2018 Apr;10(2):75-86. doi: 10.1177/1758573217728290.	No meta-analysis of interest.
115.	Pedersen JR, Andreucci A, Thorlund JB, Koes B, Møller M, Storm LK, Bricca A. Prevalence, frequency, adverse events, and reasons for analgesic use in	No meta-analysis of interest.

	youth athletes: A systematic review and meta-analysis of 44,381 athletes. J Sci Med Sport. 2022 Oct;25(10):810-819. doi: 10.1016/j.jsams.2022.08.018.	
116.	Pannone E, Abbott R. What is known about the health effects of non-steroidal anti-inflammatory drug (NSAID) use in marathon and ultraendurance running: a scoping review. BMJ Open Sport Exerc Med. 2024 Feb 2;10(1):e001846. doi: 10.1136/bmjsem-2023-001846.	No research design of interest.
117.	Pham H, Spaniol F. The Efficacy of Non-Steroidal Anti-Inflammatory Drugs in Athletes for Injury Management, Training Response, and Athletic Performance: A Systematic Review. Sports (Basel). 2024 Nov 6;12(11):302. doi: 10.3390/sports12110302.	No meta-analysis.
118.	Poretti K, Ghoddosi N, Martin J, Eddo O, Cortes N, Clark NC. The Nature of Rehabilitation Programs to Improve Musculoskeletal, Biomechanical, Functional, and Patient-Reported Outcomes in Athletes With ACL Reconstruction: A Scoping Review. Sports Health. 2024 May-Jun;16(3):390-395. doi: 10.1177/19417381231158327.	No research design of interest.
119.	Qian et al. Evaluation of diacerein and glucosamine combination therapy on osteoarthritis outcomes: a meta-analysis in an athletic population. Revista Internacional de Medicina y Ciencias de la Actividad Fisica y del Deporte. 2024;24(96).	No full text available.<br>Note: The article does not appear in the journal.
120.	Ramazzina I, Bernazzoli B, Braghieri V, Costantino C. Groin pain in athletes and non-interventional rehabilitative treatment: a systematic review. J Sports Med Phys Fitness. 2019 Jun;59(6):1001-1010. doi: 10.23736/S0022-4707.18.08879-5.	No meta-analysis.
121.	Ramirez-Campillo R, Thapa RK, Afonso J, Perez-Castilla A, Bishop C, Byrne PJ, Granacher U. Effects of Plyometric Jump Training on the Reactive Strength Index in Healthy Individuals Across the Lifespan: A Systematic Review with Meta-analysis. Sports Med. 2023 May;53(5):1029-1053. doi: 10.1007/s40279-023-01825-0.	No meta-analysis of interest.
122.	Ramos-Pastrana et al. Psychological aspects associated with ACL rehabilitation and recurrence in football players: a systematic review. Retos: nuevas tendencias en educación física, deporte y recreación. 2024;55:397-410.	No meta-analysis.

123.	Recker AJ, Waters TL, Bullock G, Rosas S, Scholten DJ 2nd, Nicholson K, Waterman BR. Biceps Tenodesis Has Greater Expected Value Than Repair for Isolated Type II SLAP Tears: A Meta-analysis and Expected-Value Decision Analysis. Arthroscopy. 2022 Oct;38(10):2887-2896.e4. doi: 10.1016/j.arthro.2022.05.005.	No meta-analysis of interest.
124.	Rogan S, Haehni M, Luijckx E, Dealer J, Reuteler S, Taeymans J. Effects of Hip Abductor Muscles Exercises on Pain and Function in Patients With Patellofemoral Pain: A Systematic Review and Meta-Analysis. J Strength Cond Res. 2019 Nov;33(11):3174-3187. doi: 10.1519/JSC.0000000000002658.	No meta-analysis of interest.<br>Note: No subgroups focused on athletes were performed.
125.	Rudisill SS, Kucharik MP, Varady NH, Martin SD. Evidence-Based Management and Factors Associated With Return to Play After Acute Hamstring Injury in Athletes: A Systematic Review. Orthop J Sports Med. 2021 Nov 29;9(11):23259671211053833. doi: 10.1177/23259671211053833.	No meta-analysis.
126.	Saini RS, Okshah A, Haneef M, Quadri SA, Chaturvedi M, Mosaddad SA, Heboyan A. Evaluating the influence of sports-induced trauma on temporomandibular disorders: A systematic review and meta-analysis. Arch Oral Biol. 2024 Dec 26;172:106168. doi: 10.1016/j.archoralbio.2024.106168.	No meta-analysis of interest.
127.	Sandler AB, Scanaliato JP, Baird MD, Dunn JC, Parnes N. Lower Reoperation and Higher Return-to-Sport Rates After Biceps Tenodesis Versus SLAP Repair in Young Patients: A Systematic Review. Arthrosc Sports Med Rehabil. 2022 Aug 26;4(5):e1887-e1895. doi: 10.1016/j.asmr.2022.07.004.	No meta-analysis of interest.
128.	Sartori et al. O impacto do consumo de álcool na recuperação muscular em homens após exercício físico: uma revisão sistemática de ensaios clínicos randomizados. Revista Brasileira de Nutrição e Esportiva. 2024;18(110):253-64.	No meta-analysis.
129.	Scheepers MS, Streak Gomersall J, Munn Z. The effectiveness of surgical versus conservative treatment for symptomatic unilateral spondylolysis of the lumbar spine in athletes: a systematic review. JBI Database System Rev Implement Rep. 2015 Apr 17;13(3):137-73. doi: 10.11124/jbisrir-2015-1926.	No meta-analysis.
130.	Senthilkumar et al. Effects of MET and Joint Mobilization on Pain Reduction and Shoulder Tightness in Athletes: A Systematic Review of Randomized	No meta-analysis.

	Controlled Trials. International Journal of Disabilities Sports and Health Sciences. 2024;7(1):261-68.	
131.	Seow D, Shimozono Y, Tengku Yusof TNB, Yasui Y, Massey A, Kennedy JG. Platelet-Rich Plasma Injection for the Treatment of Hamstring Injuries: A Systematic Review and Meta-analysis With Best-Worst Case Analysis. Am J Sports Med. 2021 Feb;49(2):529-537. doi: 10.1177/0363546520916729.	No meta-analysis of interest.
132.	Serner A, van Eijck CH, Beumer BR, Hölmich P, Weir A, de Vos RJ. Study quality on groin injury management remains low: a systematic review on treatment of groin pain in athletes. Br J Sports Med. 2015 Jun;49(12):813. doi: 10.1136/bjsports-2014-094256.	No meta-analysis.
133.	Shaari AL, Fano AN, Ferraro J, Ahmed I. Metacarpal Stress Fractures in Athletes: A Systematic Review. Hand (N Y). 2024 Jul 24:15589447241266965. doi: 10.1177/15589447241266965.	No meta-analysis.
134.	Silber ZS, Donnelly JC, Farias MJ, Lama CJ, Luther LI, Kosinski LR, Hoy AE, Gil JA. Return to Activity After Arthroscopically Assisted Triangular Fibrocartilage Complex Repair: A Systematic Review. Clin J Sport Med. 2023 May 1;33(3):270-275. doi: 10.1097/JSM.0000000000001114.	No meta-analysis.
135.	Sivrika AP, Papadamou E, Kypraios G, Lamnisos D, Georgoudis G, Stasinopoulos D. Comparability of the Effectiveness of Different Types of Exercise in the Treatment of Achilles Tendinopathy: A Systematic Review. Healthcare (Basel). 2023 Aug 11;11(16):2268. doi: 10.3390/healthcare11162268.	No meta-analysis.
136.	Stuber KJ, Bruno P, Sajko S, Hayden JA. Core stability exercises for low back pain in athletes: a systematic review of the literature. Clin J Sport Med. 2014 Nov;24(6):448-56. doi: 10.1097/JSM.0000000000000081.	No meta-analysis.
137.	Suhett LG, de Miranda Monteiro Santos R, Silveira BKS, Leal ACG, de Brito ADM, de Novaes JF, Lucia CMD. Effects of curcumin supplementation on sport and physical exercise: a systematic review. Crit Rev Food Sci Nutr. 2021;61(6):946-958. doi: 10.1080/10408398.2020.1749025.	No meta-analysis.
138.	Suso-Martí L, La Touche R, Angulo-Díaz-Parreño S, Cuenca-Martínez F. Effectiveness of motor imagery and action observation training on musculoskeletal pain intensity: A systematic review and meta-analysis. Eur J Pain. 2020 May;24(5):886-901. doi: 10.1002/ejp.1540.	No population of interest.

139.	Sutton DA, Nordin M, Côté P, Randhawa K, Yu H, Wong JJ, Stern P, Varatharajan S, Southerst D, Shearer HM, Stupar M, Chung C, Goldgrub R, Carroll LJ, Taylor-Vaisey A. The Effectiveness of Multimodal Care for Soft Tissue Injuries of the Lower Extremity: A Systematic Review by the Ontario Protocol for Traffic Injury Management (OPTIMa) Collaboration. J Manipulative Physiol Ther. 2016 Feb;39(2):95-109.e2. doi: 10.1016/j.jmpt.2016.01.004.	No meta-analysis.
140.	Swan KG Jr, Wolcott M. The athletic hernia: a systematic review. Clin Orthop Relat Res. 2007 Feb;455:78-87. doi: 10.1097/BLO.0b013e31802eb3ea.	No meta-analysis.
141.	Szabó P, Bonet S, Hetényi R, Hanna D, Kovács Z, Prisztóka G, Križalkovičová Z, Szentpéteri J. Systematic review: pain, cognition, and cardioprotection-unpacking oxytocin's contributions in a sport context. Front Physiol. 2024 Jun 10;15:1393497. doi: 10.3389/fphys.2024.1393497.	No meta-analysis.
142.	Tamaoki MJ, Lenza M, Matsunaga FT, Belloti JC, Matsumoto MH, Faloppa F. Surgical versus conservative interventions for treating acromioclavicular dislocation of the shoulder in adults. Cochrane Database Syst Rev. 2019 Oct 11;10(10):CD007429. doi: 10.1002/14651858.CD007429.pub3.	No meta-analysis of interest.<br>Note: The meta-analysis of pain only included one study.
143.	Tanos P, Christofides I, Volpin A. The effectiveness of internal fixation in the management of acute chondral fractures. A systematic review. Knee. 2022 Dec;39:216-226. doi: 10.1016/j.knee.2022.08.001.	No meta-analysis.
144.	Tognolo L, Maccarone MC, De Trane S, Scanu A, Masiero S, Fiore P. Therapeutic Exercise and Conservative Injection Treatment for Early Knee Osteoarthritis in Athletes: A Scoping Review. Medicina (Kaunas). 2022 Jan 3;58(1):69. doi: 10.3390/medicina58010069.	No research design of interest.
145.	Venn et al. The role of cannabidiol in professional sports: A scoping review. New Zealand Journal of Sports Medicine. 2020;47(2):67-75.	No research design of interest.
146.	Vij N, Fabian I, Hansen C, Kasabali AJ, Urits I, Viswanath O. Outcomes after minimally invasive and surgical management of suprascapular nerve entrapment: A systematic review. Orthop Rev (Pavia). 2022 Aug 5;14(3):37157. doi: 10.52965/001c.37157.	No meta-analysis.
147.	Wallwork SB, Braithwaite FA. Commentary on "Effectiveness of motor imagery and action observation training on musculoskeletal pain intensity:	No research design of interest.

	A systematic review and meta-analysis" by Suso-Marti et al. Eur J Pain. 2020 Apr 12. doi: 10.1002/ejp.1573.	
148.	Wareham DM, Fuller JT, Douglas TJ, Han CS, Hancock MJ. Swimming for low back pain: A scoping review. Musculoskelet Sci Pract. 2024 Jun;71:102926. doi: 10.1016/j.msksp.2024.102926.	No research design of interest.
149.	Williams S, Whatman C, Hume PA, Sheerin K. Kinesio taping in treatment and prevention of sports injuries: a meta-analysis of the evidence for its effectiveness. Sports Med. 2012 Feb 1;42(2):153-64. doi: 10.2165/11594960-000000000-00000.	No meta-analysis of interest.
150.	Winters M, Eskes M, Weir A, Moen MH, Backx FJ, Bakker EW. Treatment of medial tibial stress syndrome: a systematic review. Sports Med. 2013 Dec;43(12):1315-33. doi: 10.1007/s40279-013-0087-0.	No meta-analysis of interest.<br><br>Note: The authors did not specify in the text or forest plots which outcome has been evaluated in meta-analyses or if different outcomes have been combined in the same meta-analysis.
151.	Xiaofei et al. META-ANALYSIS OF KANGFUXIN SOLUTION COMBINED WITH PROTON PUMP INHIBITORS IN THE TREATMENT OF PEPTIC ULCER IN ATHLETIC PATIENTS. Revista Internacional de Medicina y Ciencias de la Actividad Fisica y del Deporte. 2024;24(95).	No full text available.<br><br>Note: The article does not appear in the journal.
152.	Yang CC, Hsieh MH, Ho CC, Chang YH, Shiu YJ. Effects of Caffeinated Chewing Gum on Exercise Performance and Physiological Responses: A Systematic Review. Nutrients. 2024 Oct 24;16(21):3611. doi: 10.3390/nu16213611.	No meta-analysis.

153.	Yang N, Chen S, Cui K, Li L. Kinesio taping for ankle sprain in youth athlete: A protocol for systematic review and meta-analysis. Medicine (Baltimore). 2022 Oct 21;101(42):e31222. doi: 10.1097/MD.0000000000031222.	No research design of interest.
154.	Yun, C.-G. ., An, C. S. ., & Kim, J.-L. . (2022). Effect of Exercise Program for Injured Football Players: A Systematic Review and Meta-Analysis. International Journal of Intelligent Systems and Applications in Engineering, 10(1s), 221.	No research design of interest.
155.	Zach, S., Dobersek, U., Filho, E., Inglis, V., & Tenenbaum, G. (2018). A meta-analysis of mental imagery effects on post-injury functional mobility, perceived pain, and self-efficacy. *Psychology of Sport and Exercise, 34*, 79–87.	No research design of interest.
156.	Zandonai et al. Use of analgesics in professional soccer players: A systematic review. Apunts Sports Medicine. 2023;58(219):100415.	No meta-analysis.

Manual search analysis

Study analyzed	Review article where was manually found	Reason
Mohamed AA, Zhang X, Jan Y-K. Evidence-based and adverse-effects analyses of cupping therapy in musculoskeletal and sports rehabilitation: a systematic and evidence-based review. J Back Musculoskelet Rehabil. 2023;36:3–19; doi: 10.3233/ BMR-210242.	Elmeligie et al. Effectiveness of cupping therapy for musculoskeletal pain: an umbrella review. Human Movement. 2024;25(4):28-43.	No meta-analysis.
Neal, B.S., Barton, C.J., Gallie, R., O'Halloran, P. & Morrissey, D., 2016, 'Runners with patellofemoral pain have altered biomechanics which targeted interventions can modify: A systematic review and meta-analysis', Gait and Posture 45, 69–82. https://doi.org/10.1016/j.gaitpost.2015.11.018	Kunene SH, Taukobong NP, Ramklass S. Rehabilitation approaches to anterior knee pain among runners: A scoping review. S Afr J Physiother. 2020 Jan	Included.

	27;76(1):1342. doi: 10.4102/sajp.v76i1.1342.	
Suso-Martí L, La Touche R, Angulo-Díaz-Parreño S, Cuenca-Martínez F. Effectiveness of motor imagery and action observation training on musculoskeletal pain intensity: A systematic review and meta-analysis. Eur J Pain. 2020 May;24(5):886-901. doi: 10.1002/ejp.1540.	Wallwork SB, Braithwaite FA. Commentary on "Effectiveness of motor imagery and action observation training on musculoskeletal pain intensity: A systematic review and meta-analysis" by Suso-Marti et al. Eur J Pain. 2020 Apr 12. doi: 10.1002/ejp.1573.	No population of interest.
