# Supplementary material for: Improving Methodological Quality in Meta-Analyses of Athlete Pain Interventions: An Overview of Systematic Reviews
Source: Healthcare (Basel). 2025 Oct 2;13(19):2508. doi: 10.3390/healthcare13192508 (PMC12524677; doi:10.3390/healthcare13192508)
Supplement: Supplementary file 1 [file healthcare-13-02508-s001.zip › Suppl File 3 Overlap exercise.pdf]

Supplementary file 3. Matrices of evidence and the corrected covered area (CCA) calculations for meta-analyses evaluating the effects of physical exercise on pain intensity.

$$CCA = \frac{N-r}{rc-r} = \frac{11-11}{44-11} \frac{0}{33} = 0 = 0\%$$

Note: N is the total number of original studies (including duplicates) in the meta-analyses of interest (the sum of all checked boxes in the citation matrix). Furthermore, r is the number of original studies without accounting for duplicates. Finally, c is the number of systematic reviews included in the evidence matrix (k=4). CCA = corrected covered area.

**Number of studies without accounting for duplicates**	**Primary research (references)**	**Systematic reviews where primary research appears including primary research duplicates**
1.	Purdam CR, Johnsson P, Alfredson H, Lorentzon R, Cook JL, Khan KM. A pilot study of the eccentric decline squat in the management of painful chronic patellar tendinopathy. Br J Sports Med. 2004;38:395-7.	1. Araya Quintanilla et al. 2012
2.	Visnes H, Hoskrud A, Cook J, Bahr R. No effect of eccentric training on jumper's knee in volleyball players during the competitive season a randomized clinical trial. Clin J Sport Med. 2005;15:227-34.	2. Araya Quintanilla et al. 2012
3.	Ferber R, Kendall KD, Farr L. Changes in knee biomechanics after a hipabductor strengthening protocol for runners with patellofemoral pain syndrome. J Athl Train 2011;46:142–9.	3. Neal et al. 2016

4.	Earl JE, Hoch AZ. A proximal strengthening program improves pain, function, and biomechanics in women with patellofemoral pain syndrome. Am J Sports Med 2011;39:154–63.	4. Neal et al. 2016
5.	Willy RW, Davis IS. The effect of a hip-strengthening program on mechanics during running and during a single-leg squat. J Orthop Sports Phys Ther 2011; 41:625–32	5. Neal et al. 2016
6.	Noehren B, Pohl MB, Sanchez Z, Cunningham T, Lattermann C. Proximal and distal kinematics in female runners with patellofemoral pain. Clin Biomech (Bristol Avon) 2012;27:366–71.<br><br>Noehren B, Sanchez Z, Cunningham T, McKeon PO. The effect of pain on hip and knee kinematics during running in females with chronic patellofemoral pain. Gait Posture 2012;36:596–9.	6. Neal et al. 2016
7.	Jackson JK, Shepherd TR, Kell RT. The influence of periodized resistance training on recreationally active males with chronic nonspecific low back pain. J Strength Cond Res 2011;25:242–51.	7. Thornton et al. 2021
8.	Kachanathu SJ, Zakaria AR, Sahni A, et al. Chronic low back pain in fast bowlers a comparative study of core spinal stabilization and conventional exercises. J Phys Ther Sci 2012;24:821–5	8. Thornton et al. 2021
9.	Kumar S, Sharma VP, Negi MPS. Efficacy of dynamic muscular stabilization techniques (DMST) over conventional techniques in rehabilitation of chronic low back pain. J Strength Cond Res 2009;23:2651–9.	9. Thornton et al. 2021
10.	Abouelnaga WA, Aboelnour NH. Effectiveness of active rehabilitation program on sports hernia: randomized control trial. Ann Rehabil Med. 2019;43(3):305–13.	10. Lahuerta-Martín et al. 2023

11.	Weir A, Jansen JACG, van de Port IGL, Van de Sande HBA, Tol JL, Backx FJG. Manual or exercise therapy for long-standing adductor-related groin pain: a randomised controlled clinical trial. Man Ther. 2011;16(2):148–54.	11. Lahuerta-Martín et al. 2023
