# Supplementary material for: Improving Methodological Quality in Meta-Analyses of Athlete Pain Interventions: An Overview of Systematic Reviews
Source: Healthcare (Basel). 2025 Oct 2;13(19):2508. doi: 10.3390/healthcare13192508 (PMC12524677; doi:10.3390/healthcare13192508)
Supplement: Supplementary file 1 [file healthcare-13-02508-s001.zip › Suppl File 4 Overlap manual therapy.pdf]

Supplementary file 4. Matrices of evidence and the corrected covered area (CCA) calculations for meta-analyses evaluating the effects of manual therapy techniques on pain intensity.

$$CCA = \frac{N-r}{rc-r} = \frac{6-5}{10-5} \quad \frac{1}{5} = 0.2 = 20\%$$

Note: N is the total number of original studies (including duplicates) in the meta-analyses of interest (the sum of all checked boxes in the citation matrix). Furthermore, r is the number of original studies without accounting for duplicates. Finally, c is the number of systematic reviews included in the evidence matrix (k=2). CCA = corrected covered area.

**Number of studies without accounting for duplicates**	**Primary research (references)**	**Systematic reviews where primary research appears including primary research duplicates**
1.	Chepeha, J.C.; Magee, D.J.; Bouliane, M.; Sheps, D.; Beaupre, L. Effectiveness of a Posterior Shoulder Stretching Program on University-Level Overhead Athletes: Randomized Controlled Trial. Clin. J. Sport Med. 2018, 28, 146–152.	1. Ceballos-Laita et al. 2024<br>2. Jiménez-del-Barrio et al. 2022
2.	Ilyoung, Y.; Minhyeok, K.; Jaeseop, O. The Effects of Posterior Shoulder Stretch on Rotator Cuff Strength Ratio in Adolescent Baseball Players with Scapular Dyskinesis: A Randomized Controlled Trial. Isokinet. Exerc. Sci. 2018, 26, 63–71. [CrossRef] 41. Moore, S.D.; Laudner, K.G.; Mcloda, T.A.; Shaffer, M.A. The Immediate Effects of Muscle Energy Technique on Posterior Shoulder Tightness: A Randomized Controlled Trial. J. Orthop. Sports Phys. Ther. 2011, 41, 400–407.	3. Ceballos-Laita et al. 2024

3.	Lluch, E.; Pecos-Martín, D.; Domenech-García, V.; Herrero, P.; Gallego-Izquierdo, T. Effects of an anteroposterior mobilization of the glenohumeral joint in overhead athletes with chronic shoulder pain: A randomized controlled trial. Musculoskelet. Sci. Pract. 2018, 38, 91–98.	4. Jiménez-del-Barrio et al. 2022
4.	Ceballos-Laita, L.; Pérez-Manzano, A.; Mingo-Gómez, T.; Hernando-Garijo, I.; Medrano-De-La-Fuente, R.; Estébanez-de-Miguel, E.; Jiménez-del-Barrio, S. Range of motion and muscle function on shoulder joints of young handball athletes. J. Back Musculoskelet. Rehabil. 2021, 35, 161–167.	5. Jiménez-del-Barrio et al. 2022
5.	Gharisia, O.; Lohman, E.; Daher, N.; Eldridge, A.; Shallan, A.; Jaber, H. Effect of a novel stretching technique on shoulder range of motion in overhead athletes with glenohumeral internal rotation deficits: A randomized controlled trial. BMC Musculoskelet. Disord. 2021, 22, 402. [	6. Jiménez-del-Barrio et al. 2022
