# Supplementary material for: Improving Methodological Quality in Meta-Analyses of Athlete Pain Interventions: An Overview of Systematic Reviews
Source: Healthcare (Basel). 2025 Oct 2;13(19):2508. doi: 10.3390/healthcare13192508 (PMC12524677; doi:10.3390/healthcare13192508)
Supplement: Supplementary file 1 [file healthcare-13-02508-s001.zip › Suppl File 5 Overlap electrotherapy.pdf]

Supplementary file 5. Matrices of evidence and the corrected covered area (CCA) calculations for meta-analyses evaluating the effects of electrotherapy on pain intensity.

$$CCA = \frac{N-r}{rc-r} = \frac{9-8}{16-8} = \frac{1}{8} = 0.125 = 12\%$$

Note: N is the total number of original studies (including duplicates) in the meta-analyses of interest (the sum of all checked boxes in the citation matrix). Furthermore, r is the number of original studies without accounting for duplicates. Finally, c is the number of systematic reviews included in the evidence matrix (k=2). CCA = corrected covered area.

**Number of studies without accounting for duplicates**	**Primary research (references)**	**Systematic reviews where primary research appears including primary research duplicates**
1.	de Oliveira AR, Vanin AA, Tomazoni SS, et al. Pre-exercise infrared photobiomodulation therapy (810nm) in skeletal muscle performance and postexercise recovery in humans: what is the optimal power output? Photomed Laser Surg. 2017;35:595-603	1. Luo et al. 2022
2.	Chang WD, Wu JH, Chang NJ, Lee CL, Chen S. Effects of laser acupuncture on delayed onset muscle soreness of the biceps brachii muscle: a randomized controlled trial. Evid Based Complement Alternat Med. 2019;2019:6568976.	2. Luo et al. 2022
3.	Takenori A, Ikuhiro M, Shogo U, et al. Immediate pain relief effect of low level laser therapy for sports injuries: randomized, double-blind placebo clinical trial. J Sci Med Sport. 2016;19:980-983.	3. Luo et al. 2022<br>4. Morgan et al. 2024

4.	Aver Vanin A, De Marchi T, Tomazoni SS, et al. Pre-exercise infrared low-level laser therapy (810nm) in skeletal muscle performance and postexercise recovery in humans, what is the optimal dose? A randomized, double-blind, placebocontrolled clinical trial. Photomed Laser Surg. 2016;34:473-482	5. Luo et al. 2022
5.	Stergioulas A, Stergioula M, Aarskog R, Lopes-Martins RAB, Bjordal JM. Effects of low-level laser therapy and eccentric exercises in the treatment of recreational athletes with chronic achilles tendinopathy. Am J Sports Med 36: 881–887, 2008.	6. Morgan et al. 2024
6.	Ammendolia A, Cespites M, Iocco M. Topical use of aloe gel and low-level laser therapy in overuse tendinitis of elite volleyball players: A randomized controlled trial. Sport Sci Health 12: 209–213, 2016.	7. Morgan et al. 2024
7.	Taleb EA, Albalawi HF, Mostafa MSEM, et al. Photobiomodulation and trigger band technique on groin adductor strain in athletes.Int J Health Sci 6: 1074–1086, 2022.	8. Morgan et al. 2024
8.	Verma S, Esht V, Chahal A, et al. Effectiveness of high power laser therapy on pain and isokinetic peak torque in athletes with proximal hamstring tendinopathy: A randomized trial. BioMed Res Int. 2022: 4133883, 2022.	9. Morgan et al. 2024
