# Supplementary material for: Improving Methodological Quality in Meta-Analyses of Athlete Pain Interventions: An Overview of Systematic Reviews
Source: Healthcare (Basel). 2025 Oct 2;13(19):2508. doi: 10.3390/healthcare13192508 (PMC12524677; doi:10.3390/healthcare13192508)
Supplement: Supplementary file 1 [file healthcare-13-02508-s001.zip › Suppl File 6 Overlap medication.pdf]

Supplementary file 6. Matrices of evidence and the corrected covered area (CCA) calculations for meta-analyses evaluating the effects of medication on pain intensity.

$$CCA = \frac{N-r}{rc-r} = \frac{18-18}{36-18} \quad \frac{0}{18} \quad = 0 = 0\%$$

Note: N is the total number of original studies (including duplicates) in the meta-analyses of interest (the sum of all checked boxes in the citation matrix). Furthermore, r is the number of original studies without accounting for duplicates. Finally, c is the number of systematic reviews included in the evidence matrix (k=2). CCA = corrected covered area.

**Number of studies without accounting for duplicates**	**Primary research (references)**	**Systematic reviews where primary research appears including primary research duplicates**
1.	Gosens T, Peerbooms JC, van Laar W, den Oudsten BL. Ongoing Positive Effect of Platelet-Rich Plasma Versus Corticosteroid Injection in Lateral Epicondylitis; a Double-Blind Randomized Controlled Trial With 2-year Follow-up. Am J Sports Med. 2011; 39(6): 1200-8. doi: 10.1177/0363546510397173, PMID: 21422467	1. Gholami et al. 2016
2.	Thanasas C, Papadimitriou G, Charalambidis C, Paraskevopoulos I, Papanikolaou A. Platelet-rich plasma versus autologous whole blood for the treatment of chronic lateral elbow epicondylitis: a randomized controlled clinical trial. Am J Sports Med. 2011; 39(10): 2130-4. doi: 10.1177/0363546511417113, PMID: 21813443.	2. Gholami et al. 2016

3.	Dragoo JL, Wasterlain AS, Braun HJ, Nead KT. Platelet-Rich Plasma as a Treatment for Patellar Tendinopathy A Double-Blind, Randomized Controlled Trial. Am J Sports Med. 2014; 42(3): 610-8. doi: 10.1177/0363546513518416, PMID: 24481828.	3. Gholami et al. 2016
4.	Aggarwal AK, Shashikanth V, Marwaha N. Platelet-rich plasma prevents blood loss and pain and enhances early functional outcome after total knee arthroplasty: a prospective randomised controlled study. Int Orthop 2014; 38(2): 387-95. doi: 10.1007/s00264-013-2136-6, PMID: 24114251.	4. Gholami et al. 2016
5.	Kesikburun S, Tan AK, Yılmaz B, Yaşar E, Yazıcıoğlu K. Platelet-Rich Plasma Injections in the Treatment of Chronic Rotator Cuff Tendinopathy A Randomized Controlled Trial With 1-Year Follow-up. Am J Sports Med. 2013; 41(11): 2609-16. doi: 10.1177/0363546513496542, PMID: 23893418.	5. Gholami et al. 2016
6.	Galer BS, Rowbotham M, Perander J, Devers A, Friedman E. Topical diclofenac patch relieves minor sports injury pain. J Pain Symptom Manage. 2000;19(4):287-294. doi:10.1016/ S0885-3924(00)00125-1	6. Nudo et al. 2023
7.	Predel HG, Koll R, Pabst H, et al. Diclofenac patch for topical treatment of acute impact injuries: a randomised, double blind, placebo controlled, multicentre study. Br J Sports Med. 2004;38(3):318-323. doi:10.1136/bjsm.2003.005017	7. Nudo et al. 2023
8.	Predel HG, Pabst H, Schäfer A, Voss D, Giordan N. Diclofenac patch for the treatment of acute pain caused by soft tissue injuries of limbs: a randomized, placebo-controlled clinical trial. J Sports Med Phys Fitness. 2016;56(1–2):92-99. doi:10.1136/ bjsm.2003.005017	8. Nudo et al. 2023
9.	Predel HG, Connolly MP, Bhatt A, Giannetti B. Efficacy and safety assessment of acute sports-related traumatic soft tissue injuries using a new ibuprofen medicated plaster: results from a randomized controlled clinical trial. Phys Sportsmed. 2017;45(4):418-425. doi:10.1080/00913847.2017.1382305	9. Nudo et al. 2023

10.	Predel HG, Giannetti B, Connolly MP, Lewis F, Bhatt A. Efficacy and tolerability of a new ibuprofen 200mg plaster in patients with acute sports-related traumatic blunt soft tissue injury/contusion. Postgrad Med. 2018;130(1):24-31. doi:10.1080/00325481. 2018.1401422	10. Nudo et al. 2023
11.	Wetzel D, Menke W, Dieter R, Smasal V, Giannetti B, Bulitta M. Escin/diethylammonium salicylate/heparin combination gels for the topical treatment of acute impact injuries: a randomised, double blind, placebo controlled, multicentre study. Br J Sports Med. 2002;36(3):183-188. doi:10.1136/bjsm. 36.3.183	11. Nudo et al. 2023
12.	May JJ, Lovell G, Hopkins WG. Effectiveness of 1% diclofenac gel in the treatment of wrist extensor tenosynovitis in long distance kayakers. J Sci Med Sport. 2007;10(1):59-65. doi:10.1016/j. jsams.2006.05.009	12. Nudo et al. 2023
13.	Malmgaard-Clausen NM, Jørgensen OH, Høffner R, et al. No additive clinical or physiological effects of short-term antiinflammatory treatment to physical rehabilitation in the early phase of human Achilles tendinopathy: a randomized controlled trial. Am J Sports Med. 2021;49(7):1711-1720. doi:10.1177/0363546521991903	13. Nudo et al. 2023
14.	Dupont M, Béliveau P, Thériault G. The efficacy of antiinflammatory medication in the treatment of the acutely sprained ankle. Am J Sports Med. 1987;15(1):41-45. doi:10.1177/036354658701500106	14. Nudo et al. 2023
15.	Åström M, Westlin N. No effect of piroxicam on achilles tendinopathy: a randomized study of 70 patients. Acta Orthop. 1992;63(6):631-634. doi:10.1080/17453679209169724	15. Nudo et al. 2023
16.	Reynolds JF, Noakes TD, Schwellnus MP, Windt A, Bowerbank P. Non-steroidal anti-inflammatory drugs fail to enhance healing of acute hamstring injuries treated with physiotherapy. S Afr Med J. 1995;85(6):517-522.	16. Nudo et al. 2023
17.	Giani E, Rocchi L, Tavoni A, Montanari M, Garagiola U. Telethermographic evaluation of NSAIDs in the treatment of sport sinjuries. Med Sci Sports Exerc. 1989;21(1):1-6. doi:10.1249/00005768-198902000-00001	17. Nudo et al. 2023

18.		18. Nudo et al. 2023
